# Chromosome‐level genome of *Zoysia sinica* in the intertidal zone reveals genomic insights into waterlogging stress adaptation

**DOI:** 10.1002/tpg2.70070

**Published:** 2025-07-08

**Authors:** Hyeonseon Park, Eunji Bae, Jae Gyeong Jung, Jaewook Kim, Bae Young Choi, Geungjoo Lee, Changsoo Kim, Donghwan Shim

**Affiliations:** ^1^ Department of Biological Science Chungnam National University Daejeon Republic of Korea; ^2^ Forest Biomaterials Research Center National Institute of Forest Science Jinju Republic of Korea; ^3^ Department of Biology Education Korea National University of Education Cheongju Republic of Korea; ^4^ School of Liberal Arts and Sciences Korea National University of Transportation Chungju Republic of Korea; ^5^ Department of Horticulture Chungnam National University Daejeon Republic of Korea; ^6^ Department of Smart Agriculture Systems Chungnam National University Daejeon Republic of Korea; ^7^ Department of Crop Science, College of Life Sciences Chungnam National University Daejeon Republic of Korea; ^8^ Center for Genome Engineering Institute for Basic Science Daejeon Republic of Korea

## Abstract

*Zoysia sinica* is a perennial grass that thrives in intertidal zones, even under extreme waterlogging stress. In this study, we present a high‐quality chromosome‐level genome assembly of *Z. sinica*, with a total size of 312.67 Mb. Through genome annotation, we identified 29,551 protein‐coding genes in the *Z. sinica* genome and re‐annotated 32,925, 53,226, and 53,656 genes in the previously reported *Zoysia* genomes: *Zoysia japonica*, *Zoysia matrella*, and *Zoysia pacifica*, respectively. Genome divergence analysis indicated that the *Zoysia* species diverged relatively recently, approximately 3.63 million years ago. Comparative genomic analysis revealed an expansion of ethylene response factors and identified *Z. sinica*‐specific genes related to the response to auxin and ethylene. Transcriptome data from intertidal environments with different levels of waterlogging showed significant upregulation of nitrate transporters (*NRT2.2* and *NRT2.4*) in roots and genes involved in suberin biosynthesis in shoots. Additionally, various transporters were responsive to the intertidal environment. Our study provides insights into the intertidal adaptation of *Zoysia* species and offers a foundation for the development of stress‐tolerant cultivars.

## Core Ideas

- This study presents the first chromosome‐level genome assembly of *Zoysia sinica* using Oxford Nanopore long reads and Omni‐C data, which provides a foundational genomic resource.
- We provided a re‐annotation of genomes for *Zoysia japonica*, *Zoysia matrella*, and *Zoysia pacifica*, enhancing gene annotation accuracy and comparative analyses.
- Our findings offer insights into intertidal adaptation of *Z. sinica*, highlighting genes involved in nitrogen metabolism, ethylene response, and suberin biosynthesis under waterlogging stress.

**Abbreviations**
BUSCO: benchmarking universal single‐copy ortholog
GOBP: gene ontology in terms of biological process
GSEA: gene set enrichment analysis
GWC: gravimetric water content
HIZ: high intertidal zone
IL: Inland
KEGG: Kyoto Encyclopedia of Genes and Genomes
LIZ: low intertidal zone
NES: normalized enrichment score
PCA: principal component analysis
PSG: positively selected gene
RNA‐seq: RNA sequencing
TPM: transcripts per million
WGD: whole‐genome duplication

## 1 INTRODUCTION

*Zoysia sinica* Hance is a warm‐season turfgrass belonging to the genus *Zoysia* (*Zoysia* spp. Willd.). *Zoysia* species are perennial grasses that are commonly used in golf courses, gardens, and parks, owing to their horticultural value. Therefore, *Zoysia* spp. have been a major target of breeding due to their economically important resources. One of the major phenotypic targets of breeding in *Zoysia* is the cold tolerance phenotypes (Guo et al., 2012; Jin et al., 2022; Wei et al., 2015). In the course of inbreeding the cold‐tolerant cultivar, intercross among the same *Zoysia* species and the intercross among other *Zoysia* species were both frequently utilized (Guo et al., 2012; Jin et al., 2022; Wei et al., 2015). Another phenotypic target was leaf and stolon tensile strength, which is correlated with the market value of *Zoysia* species (Choi et al., 2017; Lulli et al., 2011). *Zoysia* species were known to suffer from herbivorous bugs and fungus; thus, resistance to the pests was the target of breeding (Anderson et al., 2007; Obasa et al., 2012).

In nature, *Zoysia* species are distributed along coastlines and islands (Loch et al., 2017), and *Z. sinica* is a salt marsh turfgrass naturally distributed in Korea and Japan. The ecological distribution of *Z. sinica* ranges from mudflats that are inundated by seawater for 3 h per day to mesohaline waters consisting of soils with high salt content and little direct influence from seawater (Bang et al., 2018; J.‐S. Lee et al., 2009; S. H. Lee et al., 2018; Numata et al., 1972). *Z. sinica* is a species within the genus *Zoysia* that has adapted to waterlogged environments and is found in intertidal zones.

Although plants historically transitioned to land, recent studies have increasingly focused on their adaptations to waterlogged environments. Notably, diverse plant species in mangrove ecosystems, which thrive in coastal salt marshes and intertidal environments, have shown significant associations with ethylene response pathways and hypoxic stress response mechanisms (Liu et al., 2024; Su et al., 2022). Recently, research on the monocot mangrove species *Nypa fruticans* has reported that it maintains genomic stability over long periods through low mutation rates and a strong purifying selection (Wu et al., 2024). Key genes such as ethylene response factor VII (*ERF‐VII*), wound‐induced polypeptide 3 (*WIP3*), and UDP‐glucosyl transferase 73B3 (*UGT73B3*) play crucial roles in intertidal environments. Similarly, recent studies on *Spartina alterniflora*, a member of the Zoysieae tribe within the subfamily Chloridoideae, have demonstrated its aggressive invasion in coastal ecosystems worldwide, supported by genomic studies (S. Chen et al., 2024; Hao et al., 2024). It was reported that the gene families related to “ion transport” and “response to salt stress” have expanded and been positively selected. Such mechanisms of plant adaptation to salt and waterlogged environments are crucial for their survival in saline and waterlogged conditions. While the *Zoysia* genus, a well‐known halophyte with high resistance to abiotic stress, has been extensively studied for its salt tolerance (W. Wang et al., 2022; Zheng et al., 2022), research on its waterlogging response is lacking.

The chromosome number of *Zoysia* species has been identified as 2*n* = 40 based on experimental evidence (Forbes, 1952; Kole, 2011; Loch et al., 2017). Although the chloroplast genome *Z. sinica* has been sequenced and used in phylogenetic studies (Cheon et al., 2021), a chromosomal‐level genome assembly has not yet been reported. *Z. sinica* was classified as being closely related to *Zoysia japonica*. In the genus *Zoysia*, the genomes of *Z. japonica*, *Zoysia matrella*, and *Zoysia pacifica* were assembled in 2016 (Tanaka et al., 2016). The *Z. japonica* genome was assembled at the chromosomal level by scaffolding with the *Z. japonica* ‘El Toro’ linkage map of restriction site‐associated DNA markers (F. Wang et al., 2015). Comparative genomic analyses revealed that expanded cytochrome P450 and ABA biosynthetic gene families and many duplicated genes may contribute to salt adaptation in *Zoysia* species (W. Wang et al., 2022). To understand the differences between *Zoysia* species, the availability of a new, high‐quality genome will allow access to novel genetic variants.

Here, we present a high‐quality chromosome‐level genome assembly and landscape transcriptomic analysis across a gradient from terrestrial areas to different degrees of tidal inundation, examining both shoot and root tissues of *Z. sinica*. We analyzed the overall genomic features and gene family evolution of *Z. sinica* to infer the reasons for its adaptation to the intertidal zone and examined the gene‐level responses in actual environments through transcriptome analysis. We identified that genes associated with suberin biosynthesis in the shoots of *Z. sinica* and genes related to nitrate metabolism in the roots were significantly upregulated under waterlogging environments, suggesting these as key genes for adaptation. Our findings provide insights into the various strategies involved in the adaptation of salt marsh plants and offer a foundation for breeding waterlogging stress‐tolerant turfgrass varieties.

## 2 MATERIALS AND METHODS

### 2.1 Plant materials

*Z. sinica* was collected from Gwangpo Bay, Sacheon, South Korea. Gwangpo Bay (35.03410° N, 127.97671° E), a geographically intertidal zone, is the largest residential area of *Z. sinica* in Korea. For transcriptome research, *Z. sinica* was collected from three locations with different waterlogged environments: the high intertidal zone (HIZ) and low intertidal zone (LIZ) of Gwangpo Bay, and the Forest Biomaterials Research Center, National Institute of Forest Science (Sacheon turfgrass genetic resources conservation field) as an inland (IL) site (35°6′14.54″ N, 127°58′13.45″ E), where it is maintained under the accession number ZN2289. The collected samples included shoot (comprising leaves and sheath) and root tissues to ensure comprehensive transcriptomic coverage. Immediately after collection, the samples were flash‐frozen in liquid nitrogen and stored at −80°C until further processing.

**Core Ideas**

- This study presents the first chromosome‐level genome assembly of *Zoysia sinica* using Oxford Nanopore long reads and Omni‐C data, which provides a foundational genomic resource.
- We provided a re‐annotation of genomes for *Zoysia japonica*, *Zoysia matrella*, and *Zoysia pacifica*, enhancing gene annotation accuracy and comparative analyses.
- Our findings offer insights into intertidal adaptation of *Z. sinica*, highlighting genes involved in nitrogen metabolism, ethylene response, and suberin biosynthesis under waterlogging stress.

### 2.2 Library construction and sequencing

Genomic DNA was extracted from the leaves using Exgene Plant SV (GeneAll Biotechnology) according to the manufacturer's protocols. To extract total RNA from the shoot and root samples, the SmartGene Plant RNA Extraction Kit was used according to the manufacturer's protocol. For Oxford Nanopore long‐read sequencing, we constructed a genomic DNA library using the ONT ligation sequencing kit (SQK‐LSK110) according to the manufacturer's protocols. Sequencing was performed using flow cells (R9.4.1) on a Minion sequencer. For genomic DNA sequencing, a paired‐end library with an insert size of 150 bp was constructed using the xGen DNA Library Prep EZ UNI Kit. For RNA sequencing, mRNA was enriched using the Poly(A) RNA Selection Kit (Lexogen). The enriched mRNA was then subjected to 150‐bp paired‐end library preparation using the xGen RNA Lib Prep kit (Integrated DNA Technologies). All Illumina libraries were sequenced on the Illumina NovaSeq 6000 platform.

For the preparation of Omni‐C libraries, the Dovetail Omni‐C Kit (Dovetail Genomics) was used according to the manufacturer's instructions. Approximately 300 mg of *Z. sinica* leaf tissue was homogenized to a fine powder in liquid nitrogen. The powdered tissue was then subjected to crosslinking by adding formaldehyde to a final concentration of 37% and incubated for 10 min at room temperature. Chromatin was then digested in situ with DNase I provided in the kit, and the resulting DNA fragments were end‐repaired, ligated, and purified. The ligated DNA was then subjected to proximity ligation to create long‐range interactions, followed by reverse crosslinking to release the DNA. The resulting DNA was purified and quantified using Qubit (Thermo Fisher Scientific). The purified DNA was used to construct Omni‐C libraries using the Dovetail Library Module for Illumina (Dovetail Cat. No. 21004). Libraries were indexed using the Dovetail Dual Index Primer Set for Illumina (Dovetail Cat. No. 25005) and amplified according to the manufacturer's protocol. The quality and quantity of the libraries were assessed using Qubit 4.0 Fluorometer (Invitrogen Ltd) and 4150 TapeStation (Agilent), respectively. Sequencing of the library was performed on an Illumina NovaSeq 6000 platform with paired‐end 151‐bp reads.

### 2.3 Genome estimation, assembly, and scaffolding

The Illumina reads were cleaned using Trimmomatic v0.39 (Bolger et al., 2014). The *k*‐mer frequency analysis and estimation of genome size were performed using jellyfish v2.3.0, with the *k*‐mer size option 19 (Marçais & Kingsford, 2011), and Genomescope (Vurture et al., 2017), respectively, using Illumina reads. Nanopore reads were corrected for Illumina reads using LoRDEC, with the *k*‐mer size option 19 (Salmela & Rivals, 2014). The corrected Nanopore reads were assembled into contigs using NextDeNovo v2.5.0 with options ‐k19 and ‐w19 (Hu et al., 2024). The assembled contigs were scaffolded into 20 chromosomes using the Omni‐C data, processed with Juicer v1.6 (Durand et al., 2016) and the 3D‐DNA (v180922) pipeline (https://github.com/aidenlab/3d‐dna) (Dudchenko et al., 2017). Additionally, gaps in the genome were closed using the Nanopore reads with YAGCloser (https://github.com/merlyescalona/yagcloser), ensuring a more complete and accurate genome assembly. Finally, the chromosomal order was adjusted using the previously reported chromosome‐level genome of the closely related *Z. japonica* (F. Wang et al., 2015). The Omni‐C contact map was visualized using Juicebox. Assembly statistics were calculated using Quast v5.1.0 (Gurevich et al., 2013). To verify the genome coverage and quality of the assembled genome sequences, 4896 Poales orthologs were aligned using benchmarking universal single‐copy orthologs (BUSCOs) v5.2.2 (Manni et al., 2021). Additionally, the completeness of the genome was assessed based on 19‐mers from Illumina reads using meryl v1.3 and merqury v1.3 3 (Rhie et al., 2020). Cleaned Illumina reads were also mapped to the genome using BWA v0.7.17‐r1188 (H. Li, 2013) to further support the completeness assessment.

### 2.4 Genome annotation

We identified known and novel repetitive elements in the *Z. sinica* genome using RepeatModeler v2.0.4 (www.repeatmasker.org/RepeatModeler/) (Flynn et al., 2020) with default parameters to build a database. Prediction and masking of repeat sequences were performed using RepeatMasker v4.1.5 (http://www.repeatmasker.org/). Telomeric regions of the *Z. sinica* genome were identified using the TeloExplorer module of QuarTeT (v1.2.0) with the “‐c plant” option. For centromere prediction, the CentroMiner module was utilized, incorporating transposable element annotation data to detect tandem repeat monomers. Potential centromeric repeats were filtered based on repeat period and copy number, followed by clustering to minimize redundancy. The identified regions were visualized using RIdeogram. For gene annotation, a genome with soft‐masked repeated sequences was used. The BRAKER3 pipeline (Gabriel et al., 2024; G. Pertea & Pertea, 2020; Quinlan, 2014) was employed to annotate the protein‐coding genes of *Z. sinica* using two different hints: short‐read RNA sequencing (RNA‐seq) data and protein homology information. PRINSEQ‐lite v0.20.4 was used to trim and filter short RNA‐seq reads with the following parameters: min len 50, min qual score 10, min qual mean 20, derep 14, trim qual left 20, and trim qual right 20 (Schmieder & Edwards, 2011). Cleaned reads were aligned to the genome using HISAT2 v2.2.1 (Kim et al., 2019), and the resulting mapping files were supplied to the BRAKER3 pipeline along with protein sequences from 27 species within the class Liliopsida, downloaded from NCBI (Table S1). The hints were used for training GeneMark‐ETP and AUGUSTUS to predict genes (Bruna et al., 2024).

The functional annotation of protein‐coding genes was performed using EnTAP v1.1.1 (Hart et al., 2020). Protein sequences were analyzed against the NCBI RefSeq database (O'Leary et al., 2015) and the Uniprot database using DIAMOND (Buchfink et al., 2015) with an *e*‐value cutoff of 1$e^{-5}$. Additionally, genes were annotated with KEGG (Kyoto Encyclopedia of Genes and Genomes) terms and gene ontology terms using eggNOG‐mapper (Cantalapiedra et al., 2021; The Gene Ontology Consortium, 2014; Huerta‐Cepas et al., 2017, 2018; Kanehisa et al., 2016).

To ensure uniformity and accurate gene prediction, re‐annotation analyses for *Z. japonica*, *Z. matrella*, and *Z. pacifica* were performed using protein sequences from 27 species within the class Liliopsida. For *Z. japonica* and *Z. matrella*, transcriptome data were additionally downloaded from NCBI (Table S2) and integrated into the re‐annotation process. For *Z. pacifica*, re‐annotation was carried out using the BRAKER2 pipeline with GeneMark‐EP+ and AUGUSTUS, as no RNA‐seq data have been reported for this species (Brůna et al., 2021). The genome annotation was visualized using Circos (Krzywinski et al., 2009), and intragenomic gene synteny was detected using MCScan software with parameters –cscore 0.99 and –minspan = 30 (Tang et al., 2024).

### 2.5 Phylogenomic analysis and divergence time estimation

Genomic data for the 11 plant species were downloaded from various sources: *Arabidopsis thaliana* (TAIR10), *Brachypodium distachyon* (v3.2), *Oropetium thomaeum* (v1.0), *Sorghum bicolor* (v5.1), *Setaria italica* (v2.2), and *Zea mays* (RefGen_V4) were downloaded from Phytozome 13; *Oryza sativa* (IRGSP‐1.0) was downloaded from Ensembl (Yates et al., 2021); *S. alterniflora* was downloaded from the National Genomics Data Center under project numbers PRJCA016599 (Hao et al., 2024); and *Z. japonica*, *Z. matrella*, and *Z. pacifica* were downloaded from the Zoysia Genome Database (http://zoysia.kazusa.or.jp/) (Tanaka et al., 2016). Using OrthoFinder v2.5.5 with the ‐M msa option (Emms & Kelly, 2018, 2019), we clustered gene families for the 12 plant species. We generated a rooted species tree from a concatenated alignment of single‐copy orthologs using the STAG method in OrthoFinder. The divergence times of *Z. sinica* were inferred using this tree with MCMCtree in PAML v4.10.7, employing the JC69 model, a burn‐in of 5,000,000, a sampling frequency of 30, and 10,000,000 samples (Rannala & Yang, 2007). Calibration points were sourced from the TimeTree database (https://www.timetree.org) using nwkit (Fukushima & Pollock, 2023). The resulting phylogenetic tree with divergence times was visualized using MCMCtreeR v1.1 (Puttick, 2019).

To infer whole‐genome duplication (WGD) events, we performed protein homology analysis across seven species using DIAMOND v2.1.9 with an *e*‐value cutoff of <1$e^{-5}$ (Buchfink et al., 2015). Collinear blocks were identified using MCScanX (Y. Wang et al., 2012), where regions containing at least five homologous genes were classified as collinear. Synonymous substitution rates (Ks) values for collinear orthologous gene pairs were calculated using the add_ka_and_ks_to_collinearity.pl script implemented in MCScanX. Visualization was conducted using Python libraries, including pandas, matplotlib, numpy, and seaborn.

### 2.6 Gene family expansion and contraction analyses

We analyzed gene family evolution, including expansion and contraction, for the genomes of *Z. sinica* and 11 other plant species. All input data used the protein sequences of primary transcripts. Using the gene family clusters and phylogenetic tree topology generated by OrthoFinder, we conducted the analysis with CAFE5 v1.1 (Mendes et al., 2020). CAFE employs a random birth and death model to estimate the size of gene families at each ancestral node. The significance of gene family expansion or contraction was determined with a cutoff *p*‐value < 0.01.

### 2.7 Identification of positively selected genes (PSGs)

We conducted a selection pressure analysis on the 265 single‐copy orthologous gene sets retrieved from the genomes of seven species—*O. sativa*, *O. thomaeum*, *S. alterniflora*, *Z. matrella*, *Z. japonica*, *Z. pacifica*, and *Z. sinica*—using OrthoFinder. To prevent out‐of‐frame indels, we first aligned the translated protein sequences and then used these protein alignments to generate the codon alignments, utilizing PAL2NAL (Suyama et al., 2006) and TranslatorX (Abascal et al., 2010). The alignments were then trimmed using Gblocks (Castresana, 2000) and converted into PAML input format. For the branches of *S. alterniflora* and *Z. sinica* that inhabit intertidal zones, as well as the branch specific to *Zoysia species*, we identified PSGs (Álvarez‐Carretero et al., 2023). Subsequently, the branch‐site model (setting model = 2, Nssites = 2) was employed to determine the frequency of positive selection in each lineage. Two models were fitted: the alternative model with fix_omega = 0 and omega = 2, and the null model with fix_omega = 1 and omega = 1. These models were compared using a likelihood ratio test with 1 degree of freedom. Genes with a *p*‐value below 0.05 were classified as PSGs.

### 2.8 Gravimetric water content (GWC) and porosity measurements of soil

Soil samples were collected from the LIZ, HIZ, and the Forest Biomaterials Research Center, National Institute of Forest Science (Sacheon turfgrass genetic resources conservation field) as an IL site (35°6′14.54″ N, 127°58′13.45″ E). A 100‐mL core sampler was used to analyze soil physical properties. At each location, the top 1–2 cm of soil was removed, and 500 g samples were collected from a depth of 10 cm in five replicates. The samples were immediately sealed to prevent any changes in soil moisture before analysis. Approximately 500 g of soil was collected from each location. The GWC of the soil samples was determined following standard protocols (Robinson et al., 2008). Each soil sample was first weighed to obtain fresh weight ($W_{f}$). The samples were then dried in an oven at 70°C for 48 h to a constant weight, after which they were weighed again to determine the dry weight ($W_{d}$). GWC was calculated using the following formula:

$$\mathrm{GWC}\;\left(\%\right)=\frac{W_{f}-W_{d}}{W_{d}}\times 100$$

Soil porosity was measured using the bulk density method. The bulk density ($\rho_{b}$) was calculated as the dry weight of the soil ($W_{d}$) divided by its volume. The particle density ($\rho_{p}$) was assumed to be 2.65 g/cm^3^, a standard value for mineral soils. Soil porosity (*φ*) was calculated using the following equation:

$$\mathrm{Porosity}\;\left(\%\right)=\left(1-\frac{\rho_{b}}{\rho_{p}}\right)\times 100$$

The GWC and porosity measurements were conducted in quintuplicate for each sample location. Statistical significance between the different locations was assessed using analysis of variance (ANOVA), followed by Tukey's post hoc test, with a *p*‐value of <0.05 considered significant.

### 2.9 Gene expression analysis

For transcriptome analysis, shoot and root tissues were collected from three locations: LIZ, where roots are waterlogged during low tide; HIZ, where roots are not waterlogged during low tide; and IL (Figure 3A). The reads were cleaned using PRINSEQ‐lite v0.20.4 (Schmieder & Edwards, 2011) and Trimmomatic v0.39 (Bolger et al., 2014). The cleaned reads were then aligned to the *Z. sinica* genome using HISAT2 v2.2.1 (Kim et al., 2019). Gene expression levels were quantified with StringTie v2.2.1 based on transcripts per million (TPM) (M. Pertea et al., 2016). Principal component analysis (PCA) was performed using PtR to explore relationships between sample replicates. Differentially expressed genes (DEGs) were identified using edgeR with trimmed mean of M‐values‐normalized TPM counts (|log2(FoldChange)| > 2 and *p*‐value < 0.001). For GOBP (gene ontology in terms of biological process) and KEGG functional enrichment analysis, a BLASTp homology search was conducted with an *e*‐value cutoff of 1$e^{-5}$ between *Z. sinica* and *O. sativa* (Altschul et al., 1990). GOBP and KEGG functional enrichment analyses were performed using DAVID (Sherman et al., 2022), and the significant results (*p*‐value < 0.05) were visualized using TBtools (C. Chen et al., 2020). Gene set enrichment analysis (GSEA) was performed using GSEA v4.3.3 (Mootha et al., 2003; Subramanian et al., 2005). Expression datasets were ranked based on the signal‐to‐noise ratio across the sampling locations (LIZ, HIZ, and IL), and enrichment scores were computed for predefined gene sets. The ES values were then normalized to generate normalized enrichment scores (NESs). The statistical significance of the enrichment was assessed using 1000 permutations with the “gene set” option to account for variability in gene set composition.

### 2.10 Quantitative reverse transcriptase polymerase chain reaction (qRT‐PCR)

For RNA‐seq validation, 1 µg of total RNA was converted to cDNA using the SensiFAST cDNA Synthesis Kit (Meridian Bioscience) following the manufacturer's instructions. The synthesized cDNA was used for qRT‐PCR with the SensiFAST SYBR Lo‐ROX Kit (Meridian Bioscience) on the CFX Opus 96 Real‐Time PCR System (Bio‐Rad Laboratories, Inc.), using *HTA11* as the housekeeping gene, which exhibited uniform trimmed mean of M‐values‐normalized TPM values across all conditions. Primer information used in this study is listed in Table S3. qRT‐PCR was performed under the following conditions: denaturation at 95°C for 2 min, followed by 40 cycles of 95°C for 5 s, 63°C for 10 s, and 72°C for 20 s. The Cq values obtained from qRT‐PCR were converted to ΔΔCq values for comparative analysis with RNA‐seq data (Livak & Schmittgen, 2001). Correlation analysis between qRT‐PCR and RNA‐seq was performed using Python libraries, including pandas, numpy, and scipy.stats for statistical calculations, and matplotlib and seaborn for data visualization.

## 3 RESULTS

### 3.1 Genome sequencing and assembly

**FIGURE 1:**
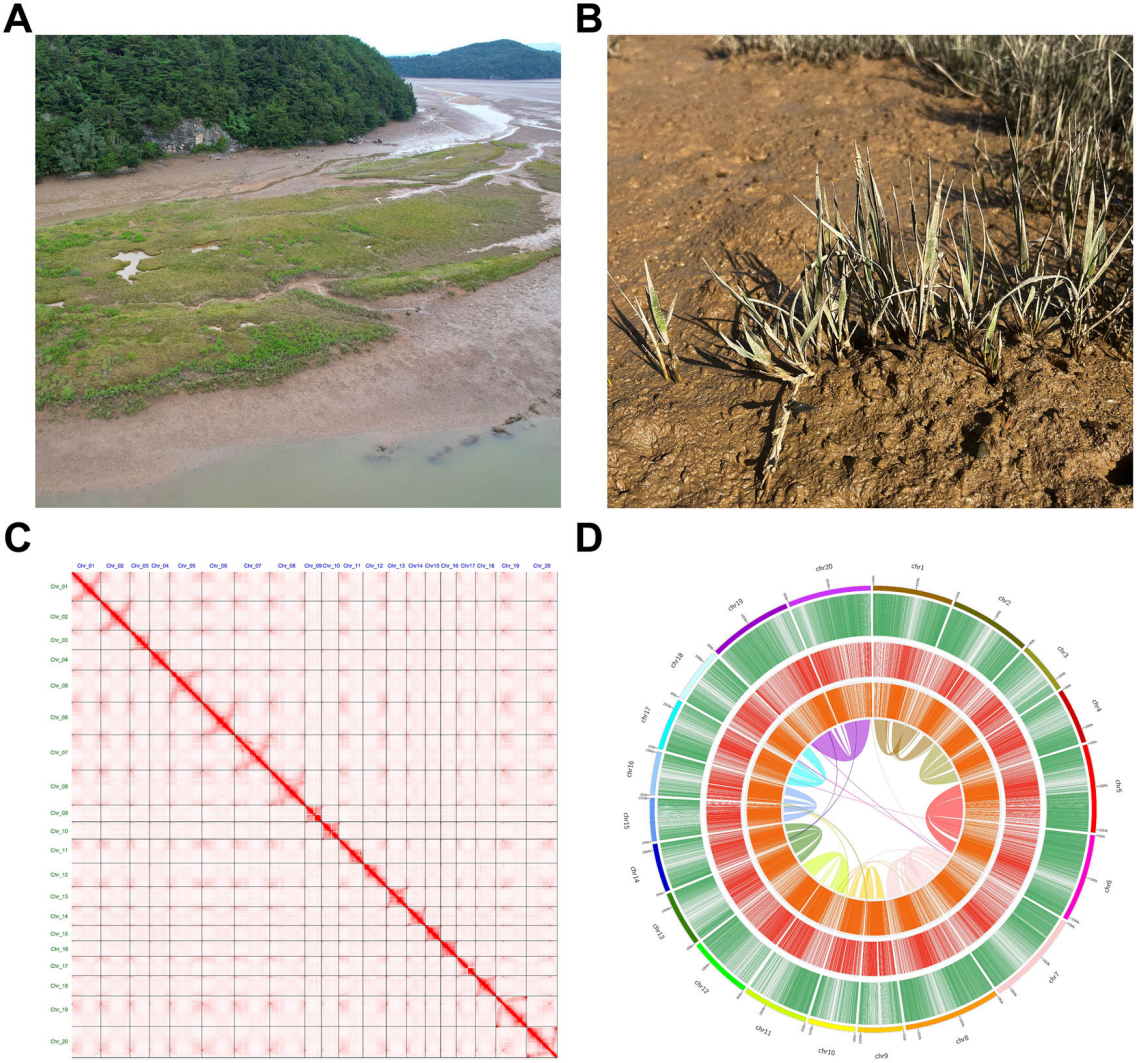
Morphological and genomic characteristics of *Z. sinica*. (A) Geographic and environmental distribution of *Z. sinica* from the intertidal zone in Gangpo Bay, Korea. (B) The appearance of *Z. sinica* growing in the low intertidal zone. (C) The Omni‐C contact map for the 20 chromosomes of *Z. sinica*. (D) Genomic feature distribution in the *Z. sinica* genome. Tracks from the outside to the inside represent protein‐coding genes, long terminal repeat (LTR) Gypsy, LTR Copia, and intragenomic synteny based on homologous genes.

*Z. sinica*, a species of perennial grasses, was collected from the Gwangpo Bay, an estuarine intertidal zone where freshwater and saltwater meet, and is the largest colony of *Z. sinica* in South Korea (Figure 1A,B). The *Z. sinica* genome size was estimated to be 328.2 Mb using a total of 19.06 Gb of Illumina reads (Table S4; Figure S1). The *k*‐mer (*k* = 19) distribution exhibited nearly one peak, indicating a low proportion of heterozygosity in the *Z. sinica* genome. To obtain long‐read sequences that covered at least an 80× coverage depth against the estimated genome size, we produced 27.27 Gb of long‐read data with 16 kb of N50 using the Oxford Nanopore Minion sequencer (Table S5).

The *Z. sinica* genome was assembled using a hybrid pipeline that combined Nanopore and Illumina reads. Illumina‐based corrected Nanopore long reads, providing an 83.8× coverage of the estimated genome size, were assembled using NextDenovo. We obtained a de novo assembled genome consisting of 73 contigs with a total length of 315.7 Mb (Table 1). Based on valid Omni‐C chromatin interaction data (Figure 1C), the contig N50 value of 10.4 Mb was significantly improved to a scaffold N50 value of 18.9 Mb. Of the 315.7 Mb contig sequences, 312.7 Mb were scaffolded and anchored onto 20 chromosomes. Gap‐closing analysis further refined the assembly, closing three gaps and resulting in the final genome with 54 remaining gaps spanning a total of 27 Kb. The 20 chromosome‐level *Z. sinica* genomes were ordered into subgenome A (odd‐numbered chromosomes) and subgenome B (even‐numbered chromosomes) using the *Z. japonica* genome as a reference (Table S6). The seven‐base telomeric repeat (CCCTAAA/TTTAGGG), which is characteristic of plants, could not be identified in the *Z. sinica* genome. However, tandem repeat monomers were identified and used to predict centromeric regions. The candidate centromeres were located at comparable positions in subgenomes A and B, suggesting structural similarity between the subgenomes (Figure S2).

**TABLE 1:** Major assembly features of the *Z. sinica* (2*n* = 4*x* = 40) genome.

Category	*Z. sinica* genome
Sequencing platform	Illumina, Oxford Nanopore
Estimated genome size (Mb)	328.2
Total contigs	73
Total contig length (Mb)	315.7
Contig N50 (Mb)	10.4
Total scaffolds	20
Total scaffold length (Mb)	312.67
Scaffold N50 (Mb)	18.9
GC (%)	43.97
Complete BUSCOs (%)	97.7
Complete and single‐copy BUSCOs (%)	73.6
Complete and duplicated BUSCOs (%)	24.1
Fragmented BUSCOs (%)	0.5
Missing BUSCOs (%)	1.8
*k*‐mer completeness (%)	86.81
Base pair QV	35.38
Annotation	
Repeat sequences (%)	42.15
Number of protein‐coding genes	29,551

Abbreviations: BUSCOs, benchmarking universal single‐copy orthologs; QV, quality value.

For genome quality assessment, the integrity of the *Z. sinica* genome was assessed by comparing its homology with the poales BUSCO genes. Out of 4896 core poales genes, 4783 (97.7%) complete genes were found in the *Z. sinica* genome, with 3604 (73.6%) being single‐copy genes and 1179 (24.1%) being duplicated genes (Table 1). Additionally, results from merqury showed that the *k*‐mer completeness was 86.81%, with a quality value of 35.38. Furthermore, Illumina reads mapping supported the high completeness of the genome, with 122,682,537 reads (97.69%) mapped, including 121,556,866 reads (96.79%) that were primary mapped. We then compared our newly assembled *Z. sinica* genome with previously reported *Zoysia* genomes including those of *Z. japonica*, *Z. matrella*, and *Z. pacifica* whose assembly sizes range from 315.7 to 563.4 Mb (Table S7). Notably, the *Z. sinica* genome has fewer contigs and a higher contig N50, with a scaffold N50 of 18.9 Mb and scaffold coverage of 99%. Excluding the contig‐level assemblies of *Z. matrella* and *Z. pacifica*, *Z. sinica* exhibits the highest genome coverage at 95.3%. Collectively, these results suggest that the *Z. sinica* genome exhibits excellent contiguity and is well‐suited for genome annotation and further studies.

### 3.2 Genome annotation of *Z. sinica* and other *Zoysia* species

To identify repetitive elements in the *Z. sinica* genome, we predicted both known and novel repetitive elements. The repetitive element prediction analysis indicated that 42.15% of the genomic sequences were annotated (Table S8). Among the repetitive elements, retroelements and DNA transposons accounted for 23.27% and 2.62% of the genome sequence, respectively. Long terminal repeat retrotransposons were predominant because Ty1/Copia and Gypsy/DIRS1 covered 6.85% and 13.23% of the genome, respectively. In addition, 15.34% of the genome was unclassified interspersed repeats.

To predict the protein‐coding genes in the masked *Z. sinica* genome, we combined ab initio‐ and homology‐based prediction strategies using the RNA‐seq data of *Z. sinica* and the protein sets of class Liliopsida (Table S1). We identified 29,551 protein‐coding genes in the *Z. sinica* genome, with an average length of 2,671 bp, 5.1 exons per gene, and an average exon length of 237 bp (Table 2). The intragenomic gene synteny analysis revealed that 10 pairs among 20 chromosomes had high collinearity based on gene homology (Figure 1D). A 5.6 Mb duplication and a 1.6 Mb translocation between the subgenomes were identified within genomic blocks based on the synteny of at least 30 consecutive genes (Table S9).

**TABLE 2:** Statistics of predicted protein‐coding genes in the *Zoysia* species.

Species	*Z. sinica* (in this study)	*Z. japonica* (Nagirizaki, v2)	*Z. matrella* (Wakaba, v2)	*Z. pacifica* (Zanpa, v2)
Protein coding genes	29,551	32,925	53,226	53,656
Mean gene length (bp)	2,671	2,625	2,361	1,786
Mean exon per transcript	5.1	5.4	4.9	3.5
Mean exon length (bp)	237	225	230	244

To ensure more reliable analysis, we re‐annotated the genomes of *Zoysia* species (*Z. japonica*, *Z. pacifica*, and *Z. matrella*) using previously reported RNA‐seq data (Table S2). We used 636 Gb of RNA‐seq data from *Z. japonica* and 95 Gb from *Z. matrella*, along with Liliopsida protein hints, for gene prediction and achieved high‐quality annotations. For *Z. pacifica*, high‐quality annotation was obtained using only Liliopsida protein hints. The quality of gene annotation was assessed through BUSCO analysis, showing significant improvement: *Z. japonica* improved from 53.8% to 95.8%, *Z. matrella* from 56.3% to 92.5%, and *Z. pacifica* from 53.5% to 75.7% (Figure S3). We predicted 32,925 genes in *Z. japonica*, 53,226 genes in *Z. matrella*, and 53,656 genes in *Z. pacifica*. The protein‐coding gene characteristics of *Z. sinica*, *Z. japonica*, and *Z. matrella* were similar (Table 2; Table S10).

### 3.3 Comparative genomic analysis of the *Z. sinica* genome

**FIGURE 2:**
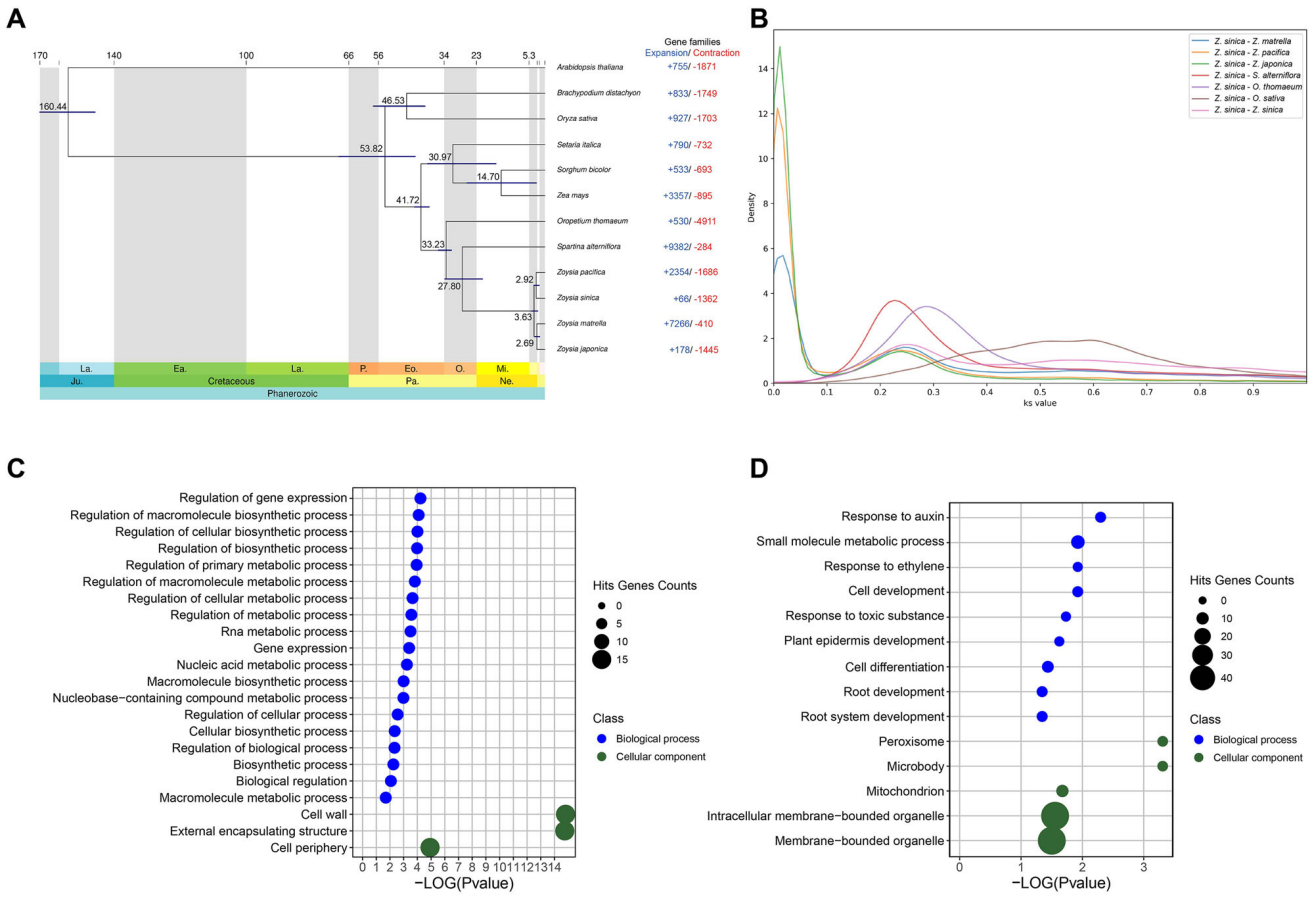
Genome and gene family evolution of *Z. sinica*. (A) The phylogenetic tree showing the divergence time of *Z. sinica* and the expansion and contraction of gene families across 12 species. (B) The Ks density distribution between *Z. sinica* and its closely related species (*O. sativa*, *O. thomaeum*, *S. alterniflora*, *Z. japonica*, *Z. matrella*, and *Z. pacifica*). (C and D) Gene ontology in terms of biological process (GOBP) enrichment analysis of the expanded gene families and *Z. sinica*‐specific genes.

To understand the evolutionary and phylogenetic relationships of *Z. sinica*, we performed a comparative genomic analysis incorporating divergence time and WGD inference. We used 473,267 representative protein‐coding genes from 12 species, which were clustered into 34,222 orthogroups. The phylogenetic analysis revealed that *Z. sinica* formed a monophyletic clade with other *Zoysia* species (*Z. japonica*, *Z. pacifica*, and *Z. matrella*), with the closest relationship observed with *Z. pacifica* (Figure 2A). Divergence time estimation indicated that the *Zoysia* species gradually diverged around 3.63 (2.42–4.45) million years ago (Mya), and they diverged from *S. alterniflora* and *O. thomaeum* around 27.8 (21.04–33.65) Mya and 33.23 (31.48–35.85) Mya, respectively. In addition, analysis of Ks among collinear gene pairs supported these WGD and speciation events of *Z. sinica* (Figure 2B). The Ks values among *Zoysia* species were very low, below 0.3, supporting the divergence time estimation (Figure 2A), which ranked *S. alterniflora*, *O. thomaeum*, and *O. sativa* in sequence. Furthermore, the Ks values of paralogs within *Z. sinica* were intermediate between the Ks values of orthologs between *Z. sinica* and *O. thomaeum* and those between *Z. sinica* and *S. alterniflora*. Additionally, the Ks values of paralogs within *Z. sinica* were similar to the Ks values of orthologs between *Z. sinica* and other *Zoysia* species. Therefore, this suggests that the WGD event in *Z. sinica* likely occurred between 27.8 and 33.2 Mya. Together, these results demonstrate that the divergence of *Z. sinica* within the *Zoysia* genus occurred relatively recently, and these findings provide important insights into the evolutionary history and polyploidization events that have influenced the genus.

To understand the genomic foundation that enabled *Z. sinica* to adapt to harsh intertidal environments, we performed a comprehensive comparative analysis that included both gene family evolution analysis and molecular evolutionary analysis to identify genes under positive selection pressure (Figure 2A). Through gene family evolution analysis, we found that *Z. sinica* exhibited expansion in only 66 gene families and contraction in 1362 gene families, with just nine gene families significantly expanded (*p*‐value < 0.01). These significantly expanded gene families were enriched in gene ontology terms related to macromolecule biosynthetic processes and cell wall (Figure 2C). Among the expanded gene families in *Z. sinica*, homologs in *O. sativa* included ethylene response factors (*OsERF11* and *OsERF17*) and RNA helicases (*OsRH6* and *OsRH12*). There were 568 *Z. sinica*‐specific genes that did not belong to any of the 12 species orthogroups, and these genes were associated with response to auxin and ethylene, response to toxic substances, and peroxisomes (Figure 2D). In parallel, our molecular evolutionary analysis across four *Zoysia* genomes revealed that *Z. sinica* harbored six PSGs, compared to nine, six, and 25 PSGs in *Z. japonica*, *Z. matrella*, and *Z. pacifica*, respectively (Figure S4; Table S11). Among them, *Z. japonica* and *Z. pacifica* shared three PSGs, indicating common selective pressures acting on these species. To further investigate the genetic basis of the adaptation of *Z. sinica* to the intertidal environment, we analyzed PSGs shared between *Z. sinica* and *S. alterniflora*, another species inhabiting intertidal environments. A total of 29 PSGs were found to be common to both species, while six PSGs were specific to *Z. sinica*, with four genes overlapping in positive selection between them (Figure S5; Table S12). The four overlapped genes were annotated to the “NmrA‐like family,” “GDSL‐like Lipase/Acylhydrolase,” “XH domain,” and “may be involved in modulation of pathogen defense and leaf cell death,” according to EGGNOG. Among the PSGs shared by *Z. sinica* and *S. alterniflora*, several key stress response genes were identified. Genes involved in nitrogen‐use efficiency (*ENOD*), osmotic regulation (*SUT*), and salt and ABA responses (*AP2/EREBP*) were detected. In addition, genes related to fungal defense, including RING finger E3 ligase and nucleotide pyrophosphatase/phosphodiesterase (*PPI*), were also under positive selection. These results indicate that gene families associated with stress responses have undergone expansion, contraction, and positive selection in *Z. sinica*, providing genomic insights into their association with intertidal environments.

### 3.4 Transcriptome analysis of the intertidal zone under distinct waterlogging conditions

We conducted a landscape transcriptomic study to examine gene expression patterns across intertidal environments with different levels of waterlogging. The physical characteristics of the soil in the intertidal environments and IL were investigated, showing significant differences in GWC among the three locations. HIZ soil had an average water content of 25.12% and LIZ soil had 22.92%, both significantly higher than the 17.66% observed in IL soil (Figure S6; Table S13 and S14).

**FIGURE 3:**
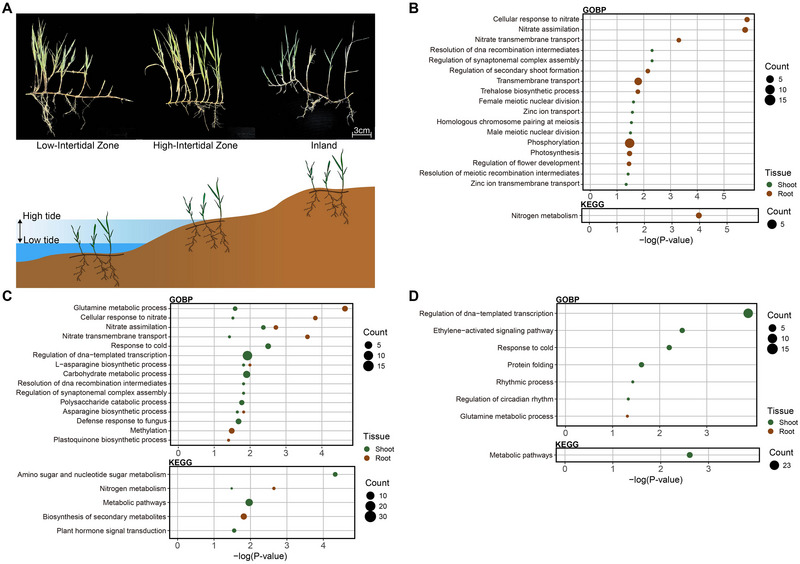
Transcriptome analysis of *Z. sinica* shoots and roots in intertidal environments. (A) Diagram of the geographic distribution of *Z. sinica*. (B–D) Gene ontology in terms of biological process (GOBP) results are shown in the upper subplots, and Kyoto Encyclopedia of Genes and Genomes (KEGG) enrichment analyses are shown in the lower subplots. The figure presents the enrichment analysis of upregulated differentially expressed genes (DEGs) in the low intertidal zone (LIZ) compared to the high intertidal zone (HIZ) (B) and inland (IL) (C). (D) Enrichment analysis of upregulated DEGs in HIZ compared to IL is also shown.

The transcriptome data from shoot and root tissues of *Z. sinica* collected from the three locations formed clusters in PCA analysis, indicating high‐quality biological replicates (Figure S7; Table S15). In shoot tissues, our analysis revealed that 84 genes were upregulated in LIZ versus HIZ, 222 in LIZ versus IL, and 156 in HIZ versus IL. In root tissues, 202 genes were upregulated in LIZ versus HIZ, 147 in LIZ versus IL, and 45 in HIZ versus IL. To interpret the biological context of the DEGs identified between locations, we performed GOBP and KEGG enrichment analyses (Figure 3). In the LIZ location, *Z. sinica* showed significant upregulation of various genes related to nitrogen metabolism in roots compared to other locations. In shoots, genes associated with nitrogen metabolism, response to cold, carbohydrate metabolic process, and polysaccharide catabolic process were upregulated compared to IL. In the HIZ location, compared to IL, genes related to Gene Ontology (GO) biological process terms such as ethylene‐activated signaling pathway and response to cold were upregulated in the shoot.

**FIGURE 4:**
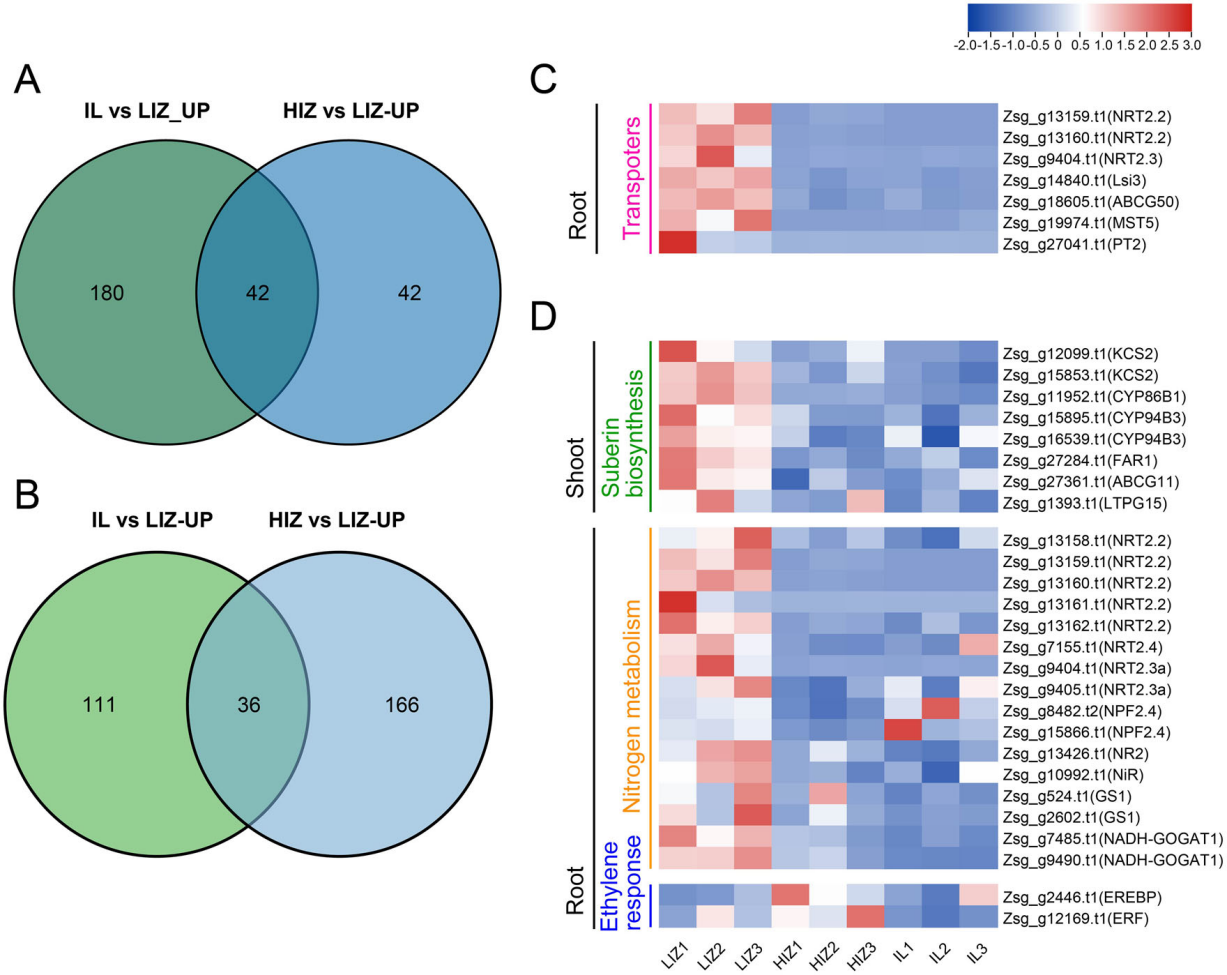
Gene expression patterns in shoot and root tissues of *Zoysia sinica* in intertidal environments. (A) Comparison of the number of upregulated differentially expressed genes (DEGs) in shoot tissues under low intertidal zone (LIZ) conditions (inland [IL] vs. LIZ conditions, high intertidal zone [HIZ] vs. LIZ conditions). (B) Comparison of the number of upregulated DEGs in root tissues under LIZ conditions (IL vs. LIZ conditions, HIZ vs. LIZ conditions). (C) Heatmap of the expression of transporter genes among the upregulated DEGs in root tissues under LIZ conditions. (D) Heatmap of the expression of genes related to suberin biosynthesis, nitrogen metabolism, and ethylene response in root and shoot tissues under waterlogging conditions.

Focusing on adaptation to persistent waterlogging stress, we identified DEGs uniquely upregulated in the LIZ environment across tidal cycles. We identified 42 DEGs specifically upregulated in the shoot (Figure 4A) and 36 DEGs in the root (Figure 4B) of the LIZ location. Notably, the DEGs specifically upregulated in the root tissue of LIZ included seven transporter genes (Figure 4C). The genes involved in suberin biosynthesis were upregulated in the shoot tissue (Figure 4D). In the root tissue, genes related to nitrate uptake (*NRT2.2* and *NRT2.4*), root–shoot nitrate translocation (*NRT2.3a*), nitrate reductase (*NR*), and glutamine synthesis (*GS* and *NADH‐GOGAT1*) were upregulated under LIZ condition. Furthermore, *EREBP* and *ERF* genes associated with ethylene response were upregulated in the root tissue under HIZ conditions. Our transcriptomic analysis across intertidal zones revealed distinct expression patterns, with a specific focus on genes uniquely upregulated in the consistently waterlogged LIZ environment. Notably, genes associated with suberin biosynthesis in the shoots and nitrate metabolism in the roots were significantly upregulated, suggesting they may play important roles in adaptation to persistent waterlogging environments.

### 3.5 GSEA of multiple abiotic stress gene sets and qRT‐PCR validation

**FIGURE 5:**
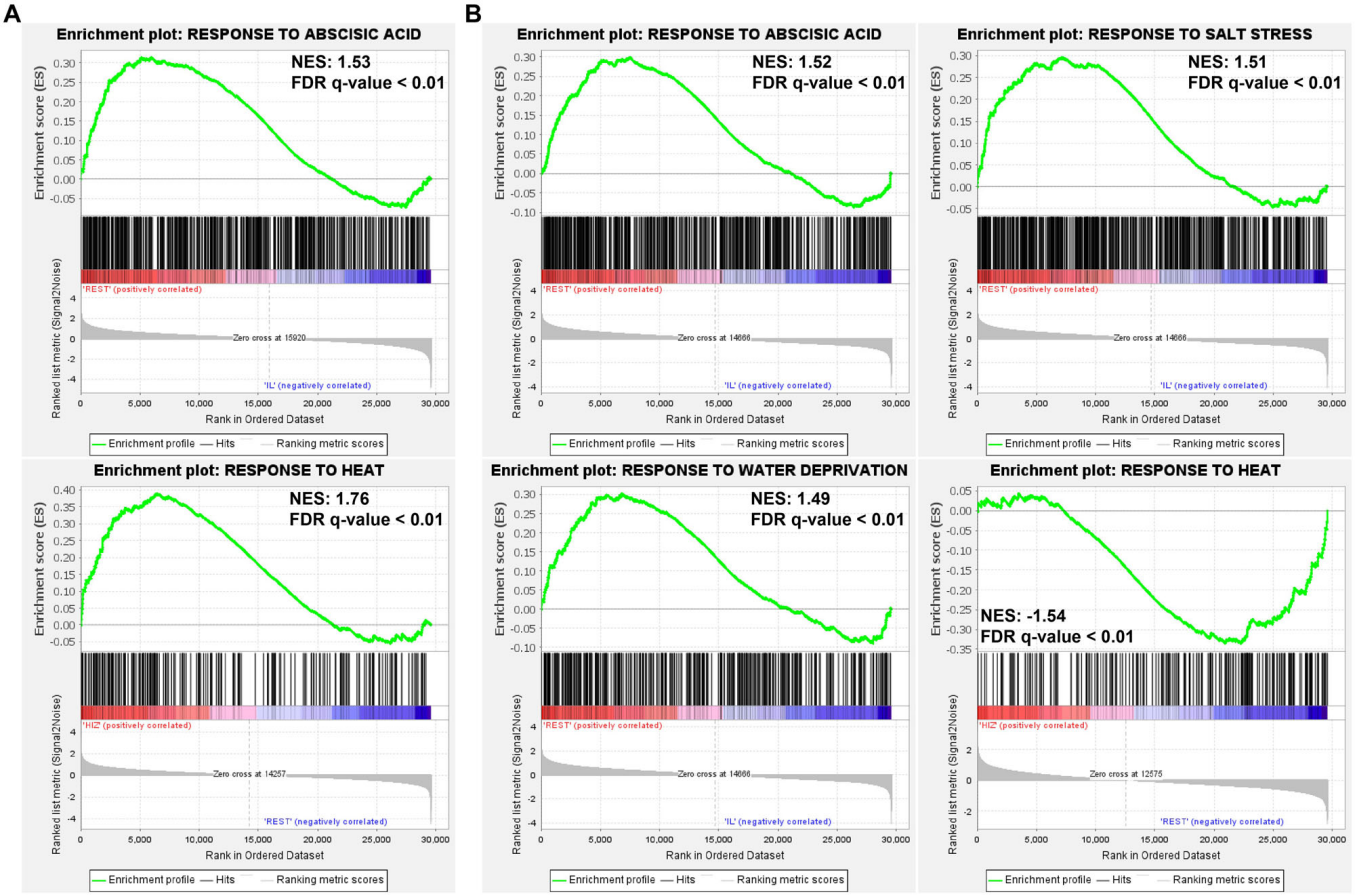
Gene set enrichment analysis (GSEA) of abiotic stress responses in *Z. sinica* under intertidal environments. The result shows significant enrichment of gene sets related to response to heat (GO:0009408), response to salt stress (GO:0009651), response to water deprivation (GO:0009414), and response to abscisic acid (GO:0009737) in (A) shoot and (B) root tissues. NES, normalized enrichment score.

To further investigate how multiple environmental factors influence the *Z. sinica* transcriptomes across locations with varying degrees of waterlogging, we performed GSEA. A total of 224 genes were assigned to response to heat (GO:0009408), 524 genes to response to salt stress (GO:0009651), 362 genes to response to water deprivation (GO:0009414), and 500 genes to response to abscisic acid (GO:0009737). In shoot tissues, GSEA revealed that the ABA response gene set was significantly enriched in intertidal conditions (LIZ and HIZ) compared to IL (false discovery rate [FDR = 0.0]) (Figure 5A). The heat response gene set was significantly enriched in HIZ relative to the combined conditions of LIZ and IL (REST) (FDR = 0.0). In root tissues, we revealed that the ABA, salt stress, and water deprivation gene sets were significantly enriched in intertidal conditions (LIZ and HIZ) compared to IL (all FDR = 0.0) (Figure 5B). The heat response gene set was significantly enriched in HIZ relative to the combined conditions of LIZ and IL (REST) (FDR = 0.0). Interestingly, unlike in the shoot tissue, where response to heat gene sets were positively enriched (NES = 1.76), the root tissue exhibited a negative correlation with NES = −1.54, suggesting tissue‐specific differences in heat stress responses. Taken together, these results suggest that both the shoot and root transcriptomes of *Z. sinica* are likely influenced by multiple abiotic environmental factors, including salt stress, water deprivation, and heat, across intertidal zones with varying waterlogging levels.

To validate the RNA‐seq results, qRT‐PCR analysis based on the ΔΔCq method showed a high correlation with the RNA‐seq data (Figure S8). Notably, five DEGs in the shoot exhibited a strong correlation between qRT‐PCR and RNA‐seq data (*R* ^2^ = 0.9162, Pearson *r* = 0.96, Spearman rho = 0.98), further supporting the reliability of the transcriptomic analysis.

## 4 DISCUSSION

In this study, we generated a high‐quality chromosome‐level genome of *Z. sinica*, a salt marsh grass adapted to saline and anoxic conditions. Compared to the previously reported chromosome‐level genome of *Z. japonica*, which was scaffolded using a linkage map and reached 81.8% scaffolding coverage (Tanaka et al., 2016), the *Z. sinica* genome exhibits significantly improved contiguity and completeness, with 95.3% scaffolding coverage (Table S7). As a result, *Z. sinica* achieved the highest genome coverage (99%) and Poales BUSCO score (95.3%) among *Zoysia* species, compared to *Z. japonica*, which had a BUSCO score of 92.9%. This high‐quality genome not only enabled reliable genomic research on the polyploid structure of *Zoysia* species but also provided an opportunity to identify marker genes involved in resilience to abiotic stresses, including waterlogging stress (Cheng et al., 2023; Njaci et al., 2023). Furthermore, it provides an important foundation for molecular breeding techniques such as marker‐assisted selection and gene editing.

To facilitate comparative genomic studies, we re‐annotated the protein‐coding genes of *Zoysia* species (*Z. japonica*, *Z. matrella*, and *Z. pacifica*) using RNA‐seq data and protein data available until recently (Table S2). This re‐annotation was confirmed to have improved integrity, as demonstrated by BUSCO analysis, ensuring high‐quality gene annotation (Figure S3). Compared to previous annotation results (Tanaka et al., 2016), the number of protein‐coding genes was reduced by half in both *Z. japonica* and *Z. matrella*, while *Z. pacifica* showed a reduction of approximately 12,000 genes. This reduction is due to improvements in the accuracy and reliability of gene annotation. Previous annotations relied on limited gene expression data, which likely led to overestimating the number of genes. In contrast, our approach combined ab initio prediction, RNA‐seq data, and protein homology information, allowing us to retain only genes that were strongly supported by multiple sources of evidence (Brůna et al., 2021; Gabriel et al., 2024). The mean gene length in the chromosome‐level genomes of *Z. sinica* and *Z. japonica* was much longer than that in the draft genomes of *Z. matrella* and *Z. pacifica* (Table 2), underscoring the importance of high‐quality genome assemblies for accurate genome annotation.

Comparative genomic analysis provided insights into the speciation timing of *Zoysia* species and the timing of the WGD event in *Z. sinica* (Figure 2A,B). It is likely that the WGD event in the common ancestor of the *Zoysia* genus occurred between its divergence from *S. alterniflora* (21.04–33.65 Mya) and *O. thomaeum* (31.48–35.85 Mya), coinciding with the speciation event from *S. alterniflora* (S. Chen et al., 2024). Paleoclimatic evidence indicates significant environmental shifts during the Miocene epoch, such as increased aridification, expansion of open grasslands, and declining atmospheric CO₂ levels (Bouchenak‐Khelladi et al., 2014; Gallaher et al., 2022). These climatic factors may have created selective pressures conducive to polyploidization, potentially enhancing adaptability to fluctuating water availability, increased salinity, and nutrient‐poor soils. Additionally, it can be inferred that *Zoysia* species underwent speciation around 2.6–3.6 Mya. This period coincided with the intensification of monsoonal patterns in East Asia, leading to alternating droughts and the expansion of salt‐affected wetlands (Dunlea et al., 2020). These climatic fluctuations likely influenced the adaptation and diversification of *Zoysia*, which is now predominantly distributed along coastal regions from temperate to tropical Southeast and Southern Asia. The inferred WGD and speciation events provide a genomic basis for understanding the geological and ecological distribution of *Zoysia* species in these dynamic environments.

The gene family evolutionary analysis provided evidence for the adaptation of *Z. sinica* to intertidal environments. The expansion of gene families related to ethylene signaling (*ERF11* and *ERF17*) (Tan et al., 2021; Zhou et al., 2016), auxin transport (*ABCB19*) (Sukumar et al., 2013), and reactive oxygen species scavenging (*APX2* and *CATB*) (Mahmood et al., 2021; Zhang et al., 2013) suggests their roles in regulating growth, oxidative stress tolerance, and root system development under periodic submergence. We identified six PSGs in *Z. sinica*, including GDSL‐like lipase (*GELP*), which is associated with suberin biosynthesis in roots (Nomberg et al., 2022; Ursache et al., 2021), and the *NmrA*‐like family gene, which plays a role in nitrogen metabolism (Mao et al., 2023) (Table S12). These genes may be associated with oxygen availability in hypoxic soil environments and contribute to nitrate‐based energy metabolism. Additionally, *Z. sinica* shares several PSGs with *S. alterniflora*, including genes related to nitrogen metabolism (*ENOD*) (BI et al., 2009) and osmotic stress tolerance (*AP2*/*EREBP*, *SUT*, and *HAK1*) (G. Chen et al., 2017; Y. Li et al., 2022; Xu et al., 2017). The presence of these genes under positive selection suggests that both species have been shaped by similar evolutionary pressures to survive in environments characterized by periodic waterlogging and salt fluctuations.

Building upon these genomic findings, transcriptome analysis examined responses of *Z. sinica* to varying soil moisture conditions across intertidal zones. The observed upregulation of nitrate uptake‐related genes in root tissues from the LIZ environment suggests their potential involvement in adaptive responses to hypoxic conditions prevalent in intertidal environments (Figure 3). A similar upregulation of nitrate transporter genes under waterlogging stress has also been observed in mangrove plants, representative species adapted to intertidal zones (Su et al., 2022). These observations, together with previous findings (G. Li et al., 2016), suggest that the upregulation of nitrate transporters, such as *NRT2.2* and *NRT2.4* in *Z. sinica*, may contribute to adaptive responses by influencing root hydraulic properties and potentially facilitating water uptake under fluctuating moisture conditions. In shoot tissues, the upregulation of suberin biosynthesis genes may help reduce radial oxygen loss and enhance adaptation to frequent submergence by maintaining cellular homeostasis and minimizing ion imbalance (Figure 4) (Kreszies et al., 2020; Ranathunge et al., 2011). Additionally, *Z. sinica* samples were collected during low tide, exposing them to aeration and a post‐submergence state. As a result, prolonged submergence followed by re‐exposure to atmospheric oxygen may lead to a water deprivation state (Figure 5) (Fukao et al., 2010; Setter et al., 2010; Shahzad et al., 2016; Yeung et al., 2018). In response, *Z. sinica* may activate ABA‐mediated signaling to facilitate submergence recovery and regulate water balance. These transcriptomic responses align with the genomic findings, suggesting that genes under positive selection, such as *GELP*, *NmrA*‐like family genes, and *ENOD*, play roles in optimizing nitrogen use and maintaining structural integrity under intertidal conditions. While our transcriptome analysis focused on responses to varying soil moisture, it is important to acknowledge that intertidal zones are complex environments where other abiotic factors, such as fluctuations in salinity and temperature, are also prevalent. These factors could act as confounding co‐stressors and potentially contribute to or modulate the observed transcriptomic profiles, and their specific impacts warrant further investigation in conjunction with waterlogging stress.

In summary, the chromosome‐level genome of *Z. sinica* provides molecular evolutionary insights into the mechanisms of adaptation and survival in intertidal environments. This study shifts the focus from the traditionally studied salt stress tolerance in zoysiagrass to a new perspective on the mechanisms of waterlogging stress responses, establishing a foundation for future research on this abiotic stress. Additionally, our landscape transcriptome study enhances our understanding of population responses to environmental variables, offering deep genomic insights with ecological significance. While these genomic and transcriptomic analyses offer significant insights, future functional studies are crucial to experimentally confirm the roles of the identified candidate genes in the adaptation of *Z. sinica* to intertidal stresses. This research provides a foundation for the development of new abiotic stress‐tolerant varieties through molecular breeding methods.

## AUTHOR CONTRIBUTIONS

**Hyeonseon Park**: Data curation; formal analysis; methodology; software; visualization; writing—original draft; writing—review and editing. **Eunji Bae**: Funding acquisition; investigation; resources; writing—original draft; writing—review and editing. **Jae Gyeong Jung**: Formal analysis; investigation; resources. **Jaewook Kim**: Methodology; software; writing—review and editing. **Bae Young Choi**: Methodology; software; visualization; writing—review and editing. **Geungjoo Lee**: Supervision. **Changsoo Kim**: Project administration. **Donghwan Shim**: Conceptualization; project administration; supervision.

## CONFLICT OF INTEREST STATEMENT

The authors declare no conflicts of interest.

## Supporting information



Figure S1. Genome size estimation of *Zoysia sinica* based on k‐mer distribution analysis; Figure S2. Chromosome Ideogram; Figure S3. BUSCO assessment of protein‐coding genes in four *Zoysia* species; Figure S4. Analysis of positively selected genes in *Zoysia* species. Figure S5. Analysis of positively selected genes in *Z. sinica* and *S. alterniflora*; Figure S6. Physical characteristics of soil in intertidal environments; Figure S7. PCA analysis of transcriptome data replicates; Figure S8. qRT‐PCR correlation analysis; Table S1. Liliopsida NCBI protein sets; Table S2. Public RNAseq data for Zoysia genus reannotation; Table S3. qRT‐PCR primer information; Table S4. The statistical information of raw and trimmed Illumina data; Table S5. The statistical information of Oxford Nanopore data; Table S6. The scaffolding statistical results of *Zoysia sinica* genome; Table S7. Statistics of the *Zoysia* genomes; Table S8. Repeat element annotation; Table S9. Macrosynteny in *Z.sinica* genome; Table S10.Zoysia genus gene annotation; Table S11. Positive‐selected genes of the *Zoysia* species; Table S12. Positive‐selected genes of *Z. sinica* and *S. alterniflora*; Table S13. Soil characteristics; Table S14. Statistics of Soil characteristics; Table S15. Statistics of RNAseq data;

## Data Availability

All sequence read files for the assembled genome have been deposited in the Sequence Read Archive database at NCBI under BioProject IDs PRJNA1149339. The Whole Genome Shotgun (WGS) project has been deposited at DDBJ/ENA/GenBank under the accession number JBGRUJ000000000 and can be accessed using this number. Additionally, the gene annotation results for four *Zoysia* species (*Z. sinica*, *Z. japonica*, *Z. matrella*, and *Z. pacifica*) have been shared via Figshare (https://doi.org/10.6084/m9.figshare.27888426.v1).
