# Supplementary figures and images for: Chromosome‐level genome of Zoysia sinica in the intertidal zone reveals genomic insights into waterlogging stress adaptation
Source: Plant Genome. 2025 Jul 8;18(3):e70070. doi: 10.1002/tpg2.70070 (PMC12238702; doi:10.1002/tpg2.70070)

# BUSCO Assessment Results

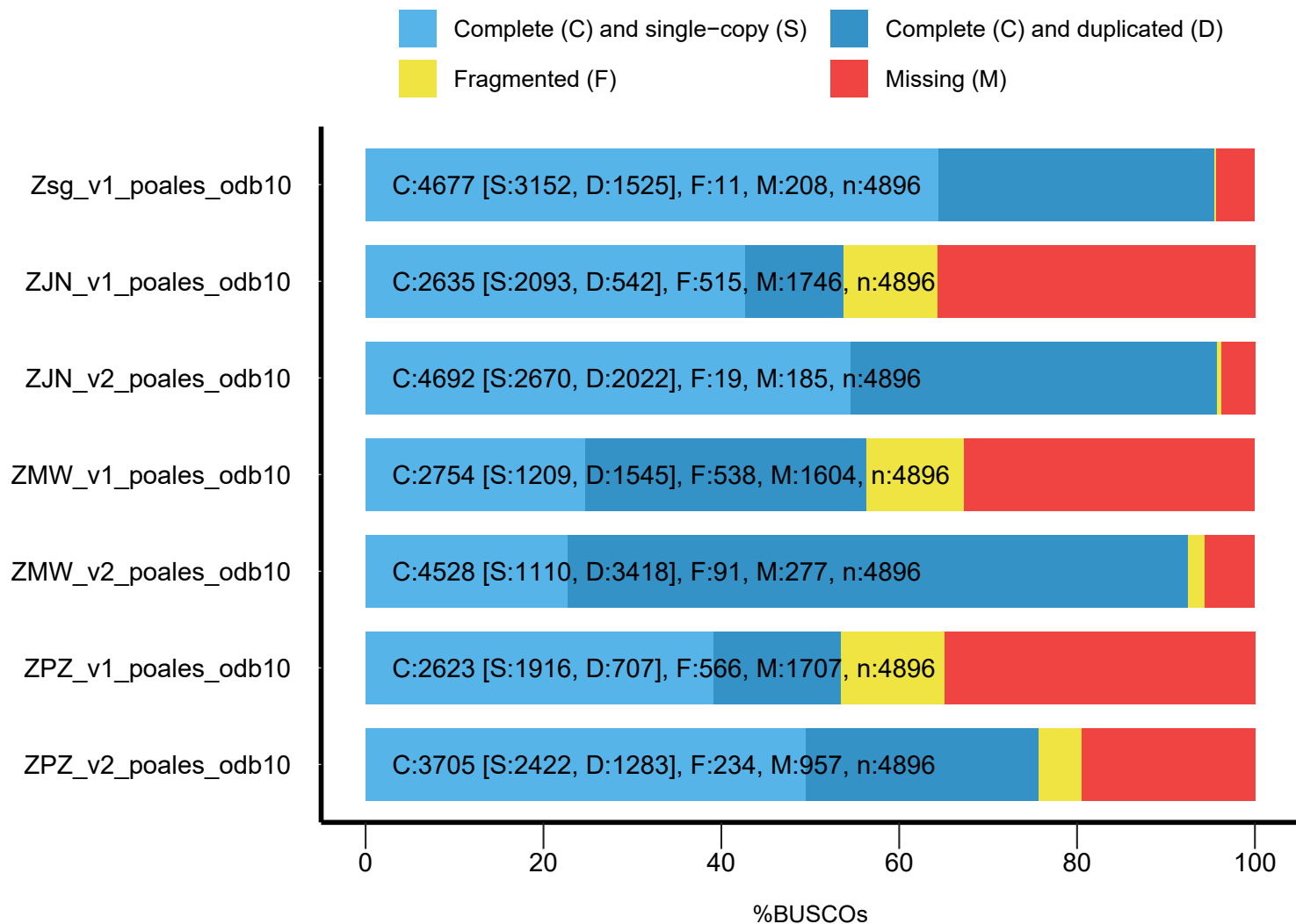

Supplement: Supplementary file 1 — Figure S1. Genome size estimation of Zoysia sinica based on k‐mer distribution analysis; Figure S2. Chromosome Ideogram; Figure S3. BUSCO assessment of protein‐coding genes in four Zoysia species; Figure S4. Analysis of positively selected genes in Zoysia species. Figure S5. Analysis of positively selected genes in Z. sinica and S. alterniflora; Figure S6. Physical characteristics of soil in intertidal environments; Figure S7. PCA analysis of transcriptome data replicates; Figure S8. qRT‐PCR correlation analysis; Table S1. Liliopsida NCBI protein sets; Table S2. Public RNAseq data for Zoysia genus reannotation; Table S3. qRT‐PCR primer information; Table S4. The statistical information of raw and trimmed Illumina data; Table S5. The statistical information of Oxford Nanopore data; Table S6. The scaffolding statistical results of Zoysia sinica genome; Table S7. Statistics of the Zoysia genomes; Table S8. Repeat element annotation; Table S9. Macrosynteny in Z.sinica genome; Table S10.Zoysia genus gene annotation; Table S11. Positive‐selected genes of the Zoysia species; Table S12. Positive‐selected genes of Z. sinica and S. alterniflora; Table S13. Soil characteristics; Table S14. Statistics of Soil characteristics; Table S15. Statistics of RNAseq data; [file TPG2-18-e70070-s001.zip › Figure_S2.busco_figure.pdf]

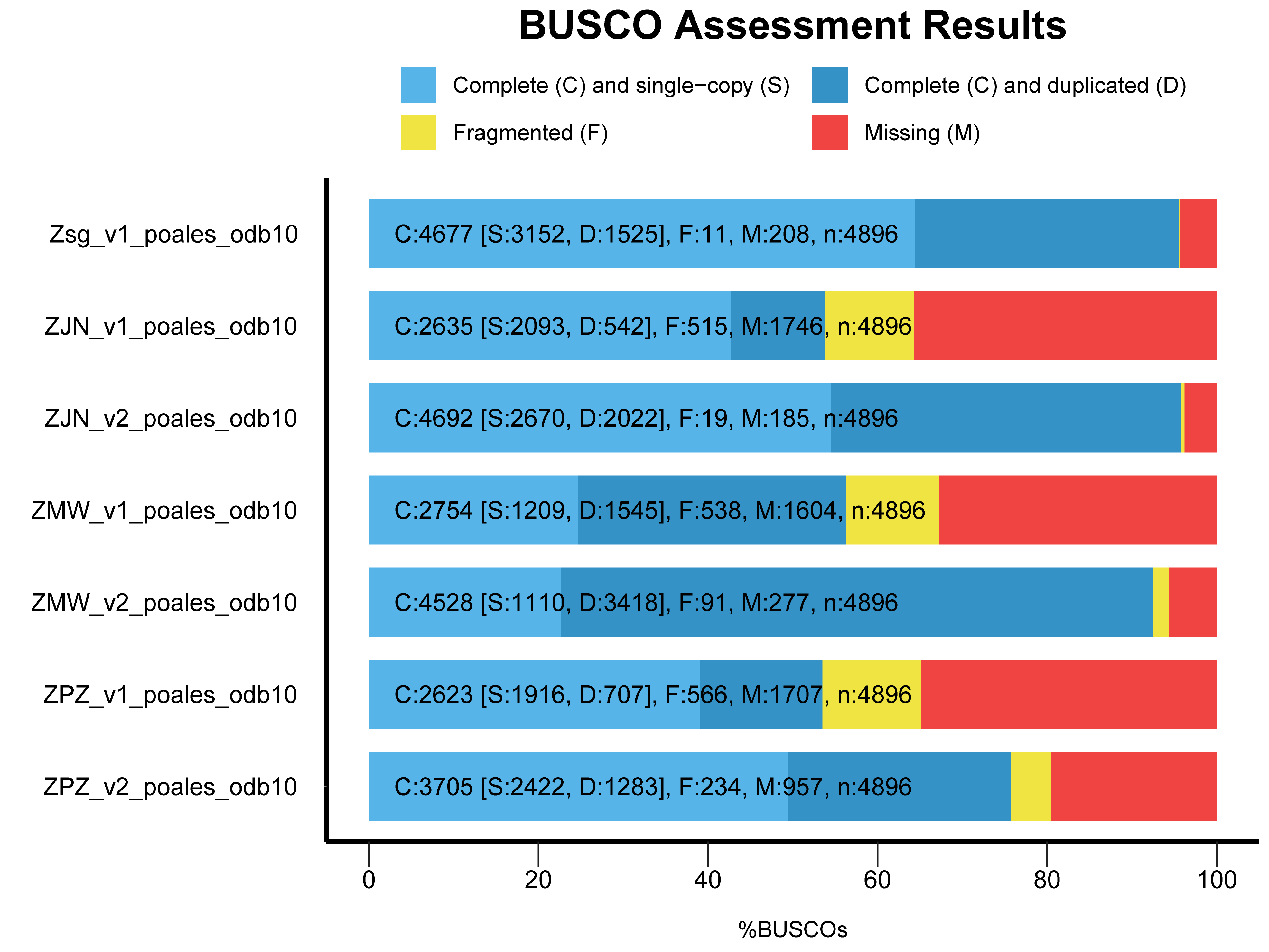

Supplement: Supplementary file 1 — Figure S1. Genome size estimation of Zoysia sinica based on k‐mer distribution analysis; Figure S2. Chromosome Ideogram; Figure S3. BUSCO assessment of protein‐coding genes in four Zoysia species; Figure S4. Analysis of positively selected genes in Zoysia species. Figure S5. Analysis of positively selected genes in Z. sinica and S. alterniflora; Figure S6. Physical characteristics of soil in intertidal environments; Figure S7. PCA analysis of transcriptome data replicates; Figure S8. qRT‐PCR correlation analysis; Table S1. Liliopsida NCBI protein sets; Table S2. Public RNAseq data for Zoysia genus reannotation; Table S3. qRT‐PCR primer information; Table S4. The statistical information of raw and trimmed Illumina data; Table S5. The statistical information of Oxford Nanopore data; Table S6. The scaffolding statistical results of Zoysia sinica genome; Table S7. Statistics of the Zoysia genomes; Table S8. Repeat element annotation; Table S9. Macrosynteny in Z.sinica genome; Table S10.Zoysia genus gene annotation; Table S11. Positive‐selected genes of the Zoysia species; Table S12. Positive‐selected genes of Z. sinica and S. alterniflora; Table S13. Soil characteristics; Table S14. Statistics of Soil characteristics; Table S15. Statistics of RNAseq data; [file TPG2-18-e70070-s001.zip › Figure_S2.busco_figure.tif]

*Zoysia sinica* PSGs

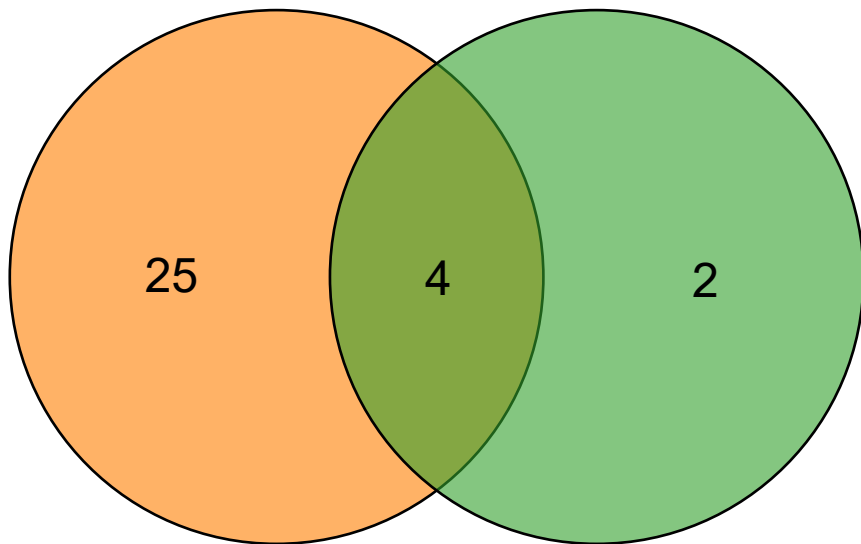

*Spartina alterniflora* & *Zoysia sinica* PSGs

Supplement: Supplementary file 1 — Figure S1. Genome size estimation of Zoysia sinica based on k‐mer distribution analysis; Figure S2. Chromosome Ideogram; Figure S3. BUSCO assessment of protein‐coding genes in four Zoysia species; Figure S4. Analysis of positively selected genes in Zoysia species. Figure S5. Analysis of positively selected genes in Z. sinica and S. alterniflora; Figure S6. Physical characteristics of soil in intertidal environments; Figure S7. PCA analysis of transcriptome data replicates; Figure S8. qRT‐PCR correlation analysis; Table S1. Liliopsida NCBI protein sets; Table S2. Public RNAseq data for Zoysia genus reannotation; Table S3. qRT‐PCR primer information; Table S4. The statistical information of raw and trimmed Illumina data; Table S5. The statistical information of Oxford Nanopore data; Table S6. The scaffolding statistical results of Zoysia sinica genome; Table S7. Statistics of the Zoysia genomes; Table S8. Repeat element annotation; Table S9. Macrosynteny in Z.sinica genome; Table S10.Zoysia genus gene annotation; Table S11. Positive‐selected genes of the Zoysia species; Table S12. Positive‐selected genes of Z. sinica and S. alterniflora; Table S13. Soil characteristics; Table S14. Statistics of Soil characteristics; Table S15. Statistics of RNAseq data; [file TPG2-18-e70070-s001.zip › Figure_S3.PSG_Venn.pdf]

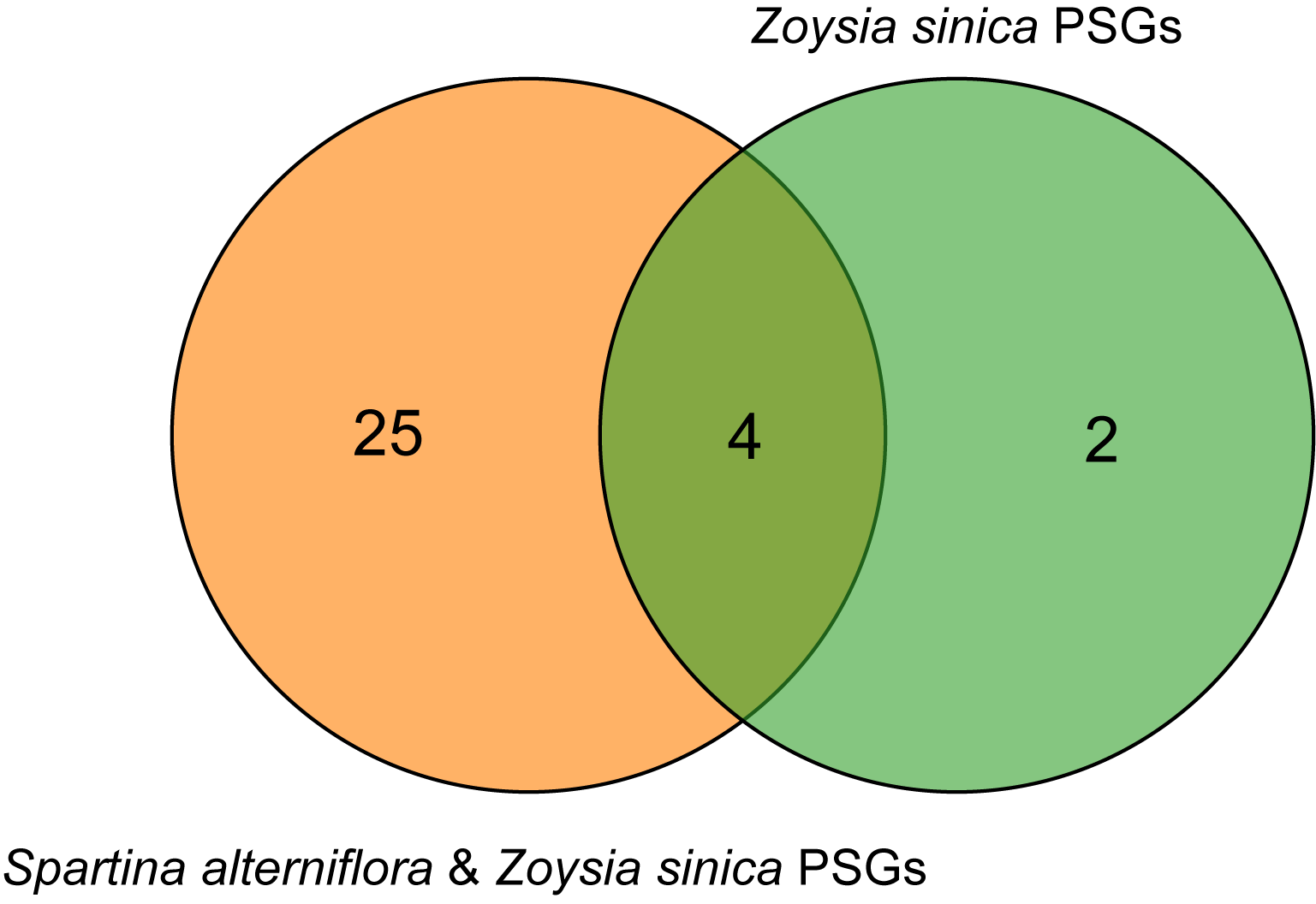

Supplement: Supplementary file 1 — Figure S1. Genome size estimation of Zoysia sinica based on k‐mer distribution analysis; Figure S2. Chromosome Ideogram; Figure S3. BUSCO assessment of protein‐coding genes in four Zoysia species; Figure S4. Analysis of positively selected genes in Zoysia species. Figure S5. Analysis of positively selected genes in Z. sinica and S. alterniflora; Figure S6. Physical characteristics of soil in intertidal environments; Figure S7. PCA analysis of transcriptome data replicates; Figure S8. qRT‐PCR correlation analysis; Table S1. Liliopsida NCBI protein sets; Table S2. Public RNAseq data for Zoysia genus reannotation; Table S3. qRT‐PCR primer information; Table S4. The statistical information of raw and trimmed Illumina data; Table S5. The statistical information of Oxford Nanopore data; Table S6. The scaffolding statistical results of Zoysia sinica genome; Table S7. Statistics of the Zoysia genomes; Table S8. Repeat element annotation; Table S9. Macrosynteny in Z.sinica genome; Table S10.Zoysia genus gene annotation; Table S11. Positive‐selected genes of the Zoysia species; Table S12. Positive‐selected genes of Z. sinica and S. alterniflora; Table S13. Soil characteristics; Table S14. Statistics of Soil characteristics; Table S15. Statistics of RNAseq data; [file TPG2-18-e70070-s001.zip › Figure_S3.PSG_Venn.tif]

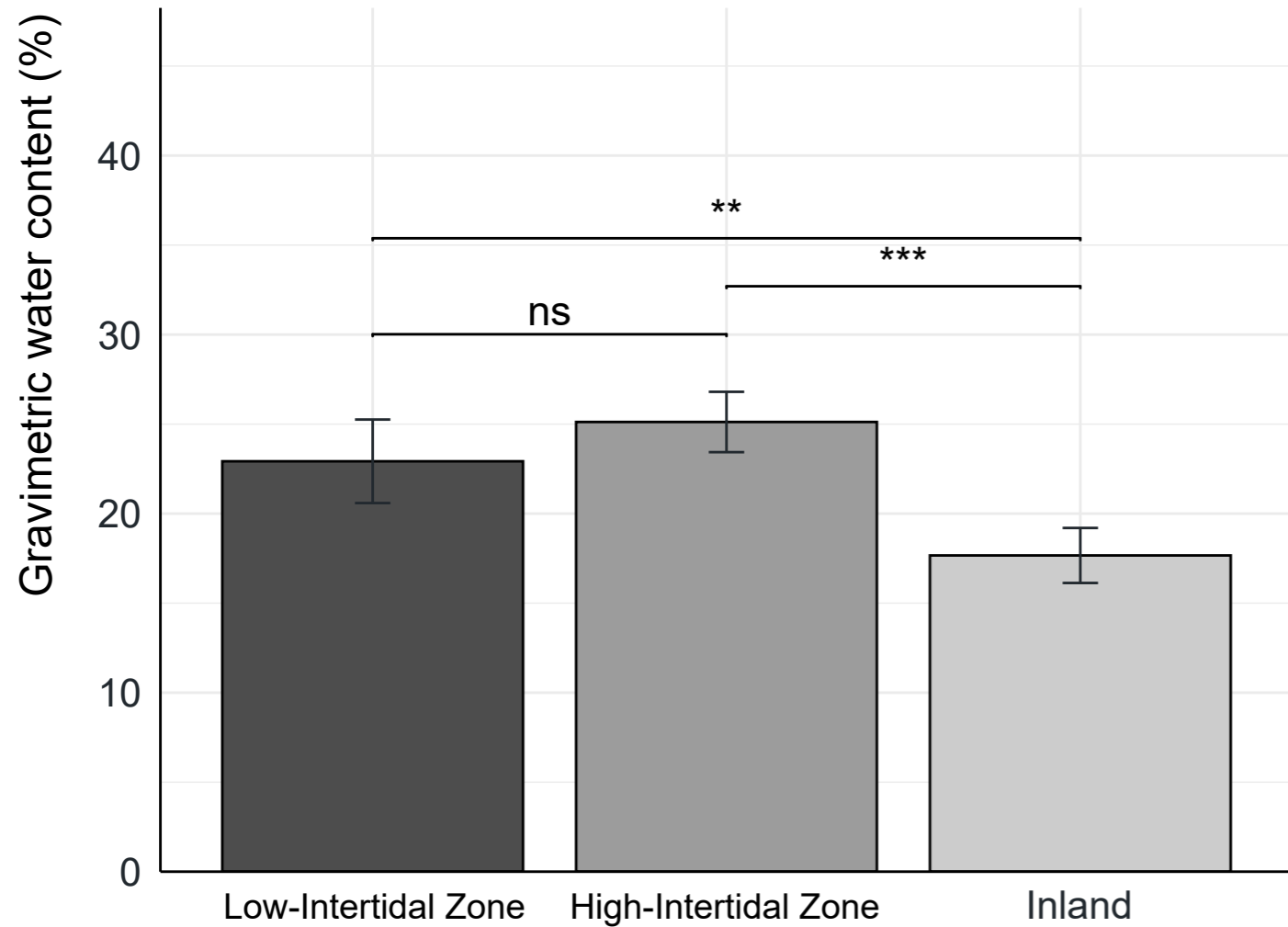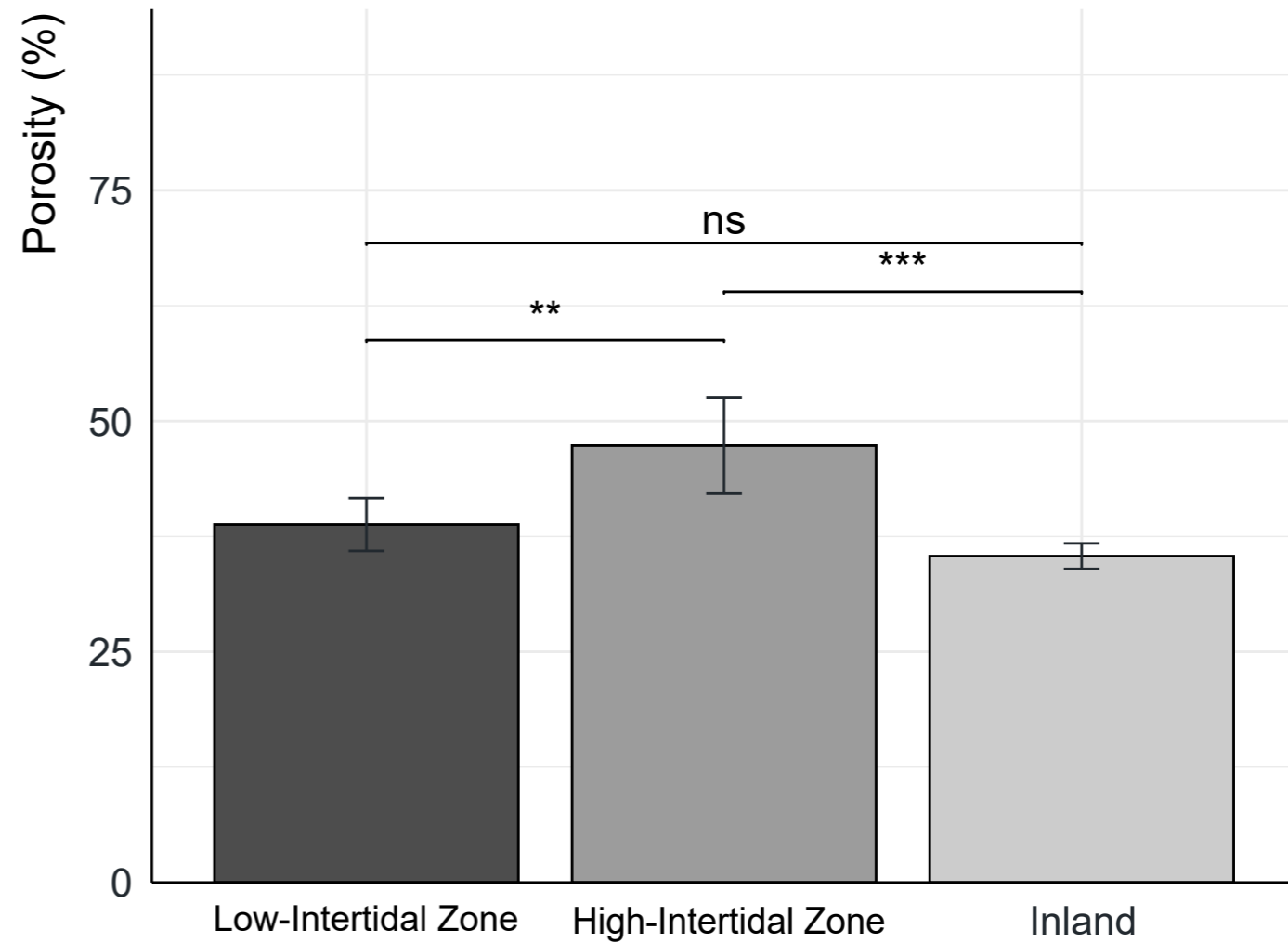

Supplement: Supplementary file 1 — Figure S1. Genome size estimation of Zoysia sinica based on k‐mer distribution analysis; Figure S2. Chromosome Ideogram; Figure S3. BUSCO assessment of protein‐coding genes in four Zoysia species; Figure S4. Analysis of positively selected genes in Zoysia species. Figure S5. Analysis of positively selected genes in Z. sinica and S. alterniflora; Figure S6. Physical characteristics of soil in intertidal environments; Figure S7. PCA analysis of transcriptome data replicates; Figure S8. qRT‐PCR correlation analysis; Table S1. Liliopsida NCBI protein sets; Table S2. Public RNAseq data for Zoysia genus reannotation; Table S3. qRT‐PCR primer information; Table S4. The statistical information of raw and trimmed Illumina data; Table S5. The statistical information of Oxford Nanopore data; Table S6. The scaffolding statistical results of Zoysia sinica genome; Table S7. Statistics of the Zoysia genomes; Table S8. Repeat element annotation; Table S9. Macrosynteny in Z.sinica genome; Table S10.Zoysia genus gene annotation; Table S11. Positive‐selected genes of the Zoysia species; Table S12. Positive‐selected genes of Z. sinica and S. alterniflora; Table S13. Soil characteristics; Table S14. Statistics of Soil characteristics; Table S15. Statistics of RNAseq data; [file TPG2-18-e70070-s001.zip › Figure_S4.Soil_Characteristics.pdf]

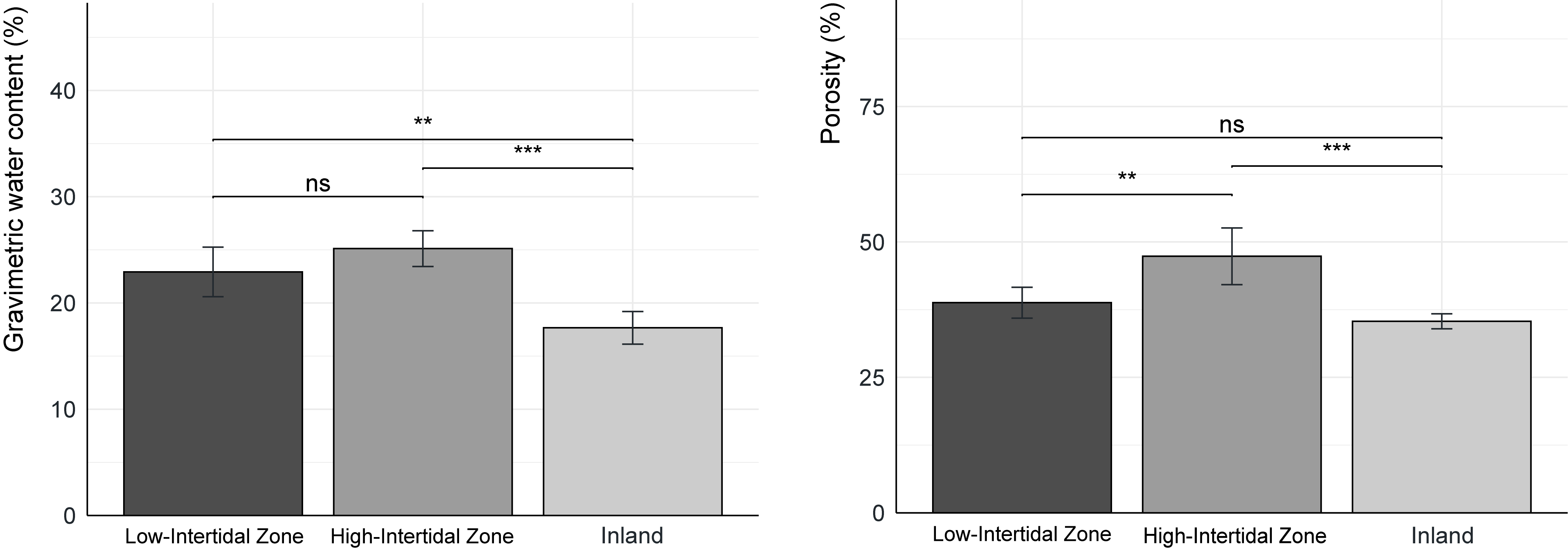

Supplement: Supplementary file 1 — Figure S1. Genome size estimation of Zoysia sinica based on k‐mer distribution analysis; Figure S2. Chromosome Ideogram; Figure S3. BUSCO assessment of protein‐coding genes in four Zoysia species; Figure S4. Analysis of positively selected genes in Zoysia species. Figure S5. Analysis of positively selected genes in Z. sinica and S. alterniflora; Figure S6. Physical characteristics of soil in intertidal environments; Figure S7. PCA analysis of transcriptome data replicates; Figure S8. qRT‐PCR correlation analysis; Table S1. Liliopsida NCBI protein sets; Table S2. Public RNAseq data for Zoysia genus reannotation; Table S3. qRT‐PCR primer information; Table S4. The statistical information of raw and trimmed Illumina data; Table S5. The statistical information of Oxford Nanopore data; Table S6. The scaffolding statistical results of Zoysia sinica genome; Table S7. Statistics of the Zoysia genomes; Table S8. Repeat element annotation; Table S9. Macrosynteny in Z.sinica genome; Table S10.Zoysia genus gene annotation; Table S11. Positive‐selected genes of the Zoysia species; Table S12. Positive‐selected genes of Z. sinica and S. alterniflora; Table S13. Soil characteristics; Table S14. Statistics of Soil characteristics; Table S15. Statistics of RNAseq data; [file TPG2-18-e70070-s001.zip › Figure_S4.Soil_Characteristics.tif]

**A**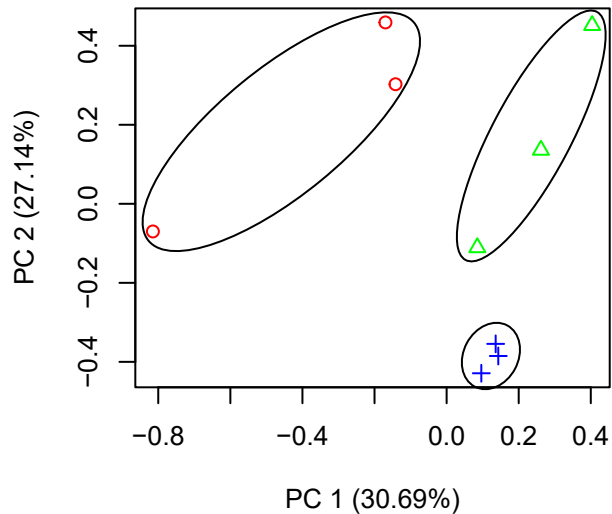**B**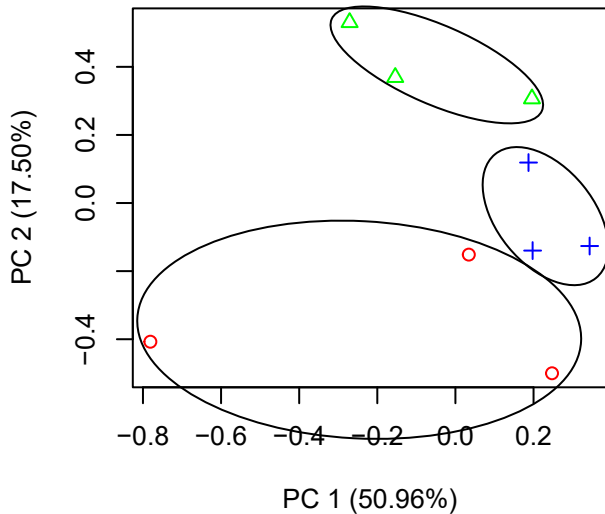

○ Inland\_Shoot

+ LIZ\_Shoot

△ HIZ\_Shoot

○ Inland\_Root

+ LIZ\_Root

△ HIZ\_Root

Supplement: Supplementary file 1 — Figure S1. Genome size estimation of Zoysia sinica based on k‐mer distribution analysis; Figure S2. Chromosome Ideogram; Figure S3. BUSCO assessment of protein‐coding genes in four Zoysia species; Figure S4. Analysis of positively selected genes in Zoysia species. Figure S5. Analysis of positively selected genes in Z. sinica and S. alterniflora; Figure S6. Physical characteristics of soil in intertidal environments; Figure S7. PCA analysis of transcriptome data replicates; Figure S8. qRT‐PCR correlation analysis; Table S1. Liliopsida NCBI protein sets; Table S2. Public RNAseq data for Zoysia genus reannotation; Table S3. qRT‐PCR primer information; Table S4. The statistical information of raw and trimmed Illumina data; Table S5. The statistical information of Oxford Nanopore data; Table S6. The scaffolding statistical results of Zoysia sinica genome; Table S7. Statistics of the Zoysia genomes; Table S8. Repeat element annotation; Table S9. Macrosynteny in Z.sinica genome; Table S10.Zoysia genus gene annotation; Table S11. Positive‐selected genes of the Zoysia species; Table S12. Positive‐selected genes of Z. sinica and S. alterniflora; Table S13. Soil characteristics; Table S14. Statistics of Soil characteristics; Table S15. Statistics of RNAseq data; [file TPG2-18-e70070-s001.zip › Figure_S5.Transcriptome_PCA_plot.pdf]

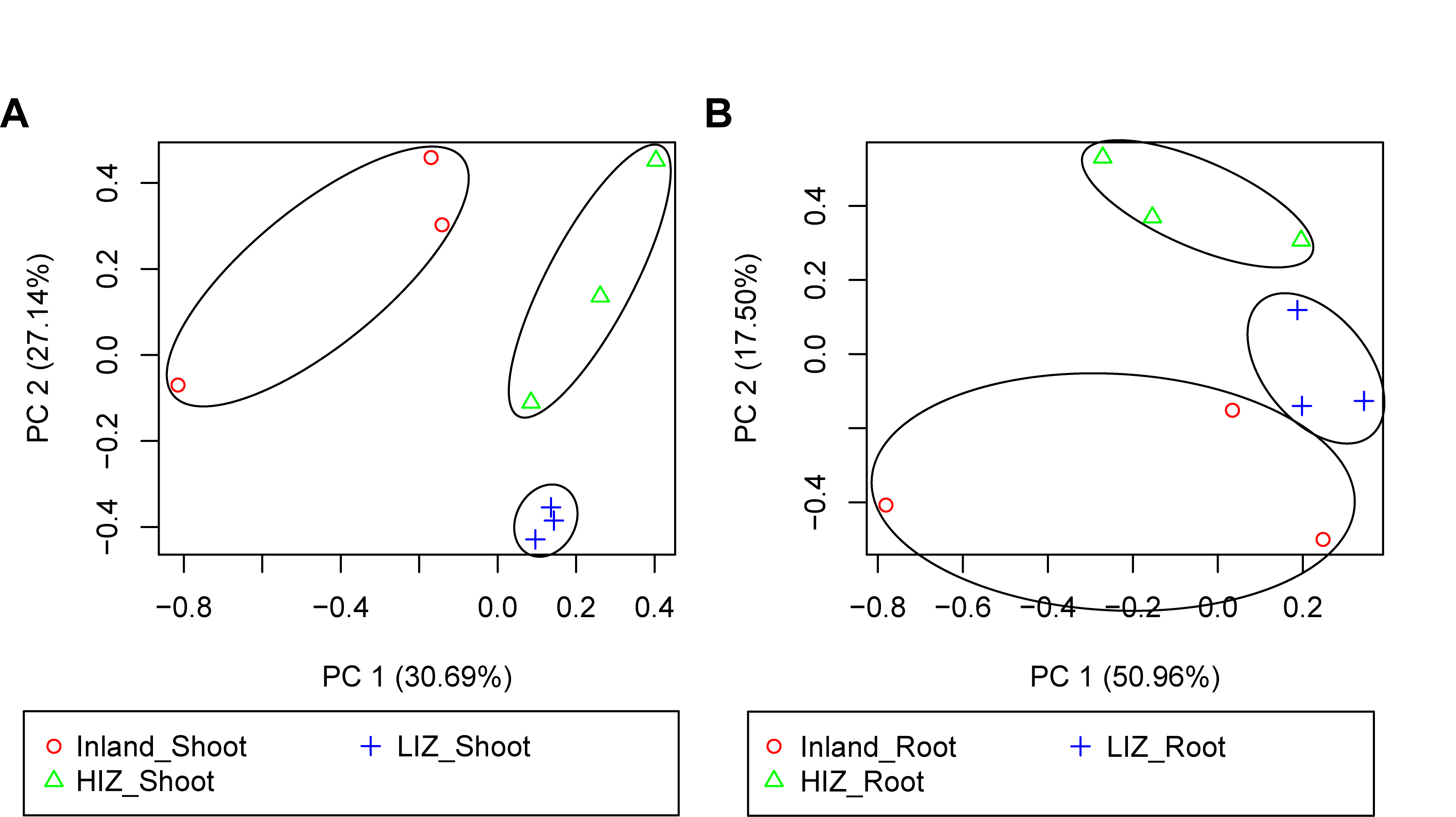

Supplement: Supplementary file 1 — Figure S1. Genome size estimation of Zoysia sinica based on k‐mer distribution analysis; Figure S2. Chromosome Ideogram; Figure S3. BUSCO assessment of protein‐coding genes in four Zoysia species; Figure S4. Analysis of positively selected genes in Zoysia species. Figure S5. Analysis of positively selected genes in Z. sinica and S. alterniflora; Figure S6. Physical characteristics of soil in intertidal environments; Figure S7. PCA analysis of transcriptome data replicates; Figure S8. qRT‐PCR correlation analysis; Table S1. Liliopsida NCBI protein sets; Table S2. Public RNAseq data for Zoysia genus reannotation; Table S3. qRT‐PCR primer information; Table S4. The statistical information of raw and trimmed Illumina data; Table S5. The statistical information of Oxford Nanopore data; Table S6. The scaffolding statistical results of Zoysia sinica genome; Table S7. Statistics of the Zoysia genomes; Table S8. Repeat element annotation; Table S9. Macrosynteny in Z.sinica genome; Table S10.Zoysia genus gene annotation; Table S11. Positive‐selected genes of the Zoysia species; Table S12. Positive‐selected genes of Z. sinica and S. alterniflora; Table S13. Soil characteristics; Table S14. Statistics of Soil characteristics; Table S15. Statistics of RNAseq data; [file TPG2-18-e70070-s001.zip › Figure_S5.Transcriptome_PCA_plot.tif]
